# Exosome-orchestrated network in gastric cancer: mechanisms, immune regulation, biomarkers and therapeutic vehicles

**DOI:** 10.3389/fcell.2026.1883199

**Published:** 2026-08-06

**Authors:** Yiyang Wang, Lei Gao, Yongkang Zhou, Lin Jiang, Zhengye Xue, Wenjun Zhu, Xuandong Ge, Wenrong Xu, Jiajia Jiang, Xueyan Zang

**Affiliations:** 1 Jiangsu Key Laboratory of Medical Science and Laboratory Medicine, School of Medicine, Jiangsu University, Zhenjiang, Jiangsu, China; 2 Aoyang Cancer Institute, Affiliated Aoyang Hospital of Jiangsu University, Zhangjiagang, Jiangsu, China

**Keywords:** diagnostic biomarkers, engineered exosomes, exosomes, extracellular vesicles, gastric cancer, immune evasion, targeted therapy, tumor microenvironment

## Abstract

Gastric cancer (GC) is a malignancy with high global incidence and mortality, and its poor prognosis and therapeutic failure are largely attributable to a complex tumor microenvironment. Extracellular vesicles (EVs) mediate intercellular molecular communication and are deeply involved in the initiation and progression of GC. Here, we focus on exosomes, the most thoroughly studied EV subtype (30–150 nm), and examine their biological roles in GC and their potential for clinical translation. We construct an exosome-derived molecular network of GC progression across six dimensions: promoting tumor cell proliferation, orchestrating an immunosuppressive microenvironment, inducing tumor angiogenesis, remodeling the extracellular matrix, guiding metastasis, and mediating drug resistance. In this review, we systematically summarize exosome-derived non-coding RNAs and proteins as biomarkers for early detection and prognosis of GC. Moreover, emerging strategies targeting exosomes or employing exosomes as delivery vehicles are discussed. Finally, we address current challenges in exosome research and propose future directions. This review aims to serve as a reference for basic research and clinical translation in GC, emphasizing that deciphering exosome-mediated intercellular dialogue is essential for understanding the molecular underpinnings of tumor progression and developing novel intervention strategies.

## 1 Introduction

Gastric cancer (GC) is intractable not merely because of what individual cancer cells do, but because it behaves as a complex ecosystem. Tumor cells maintain continuous molecular crosstalk with the surrounding stroma, including fibroblasts, immune infiltrates, and endothelial cells, all of which shape the tumor microenvironment. Extracellular vesicles (EVs) serve as key mediators of this dialogue. With a diameter of 30–150 nm, exosomes represent the most thoroughly studied EV subtype. Exosomes package a diverse payload of proteins, lipids, and nucleic acids. They engage recipient cells via ligand–receptor binding, direct membrane fusion, or endocytic uptake (Fu et al., 2019). In GC, exosomes relay signals between tumor cells and extend this dialogue to include immune cells and fibroblasts, thereby forming a multicellular communication network. Thus, they contribute to tumor proliferation, immune evasion, angiogenesis, stromal remodeling, invasion, metastasis, and therapeutic resistance (Kalluri and McAndrews, 2023).

*Helicobacter pylori*, a key driver of GC, sheds outer membrane vesicles (OMVs), bacterial-derived structures that are biogenically and biologically distinct from eukaryotic exosomes, loaded with CagA, VacA, and GroEL. These vesicle-borne virulence factors fuel the infection-to-inflammation-to-cancer sequence (Wang J. et al., 2025). Thus, OMVs from *H. pylori* and exosomes from host cells represent two distinct but cooperating vesicle systems that jointly shape the GC microenvironment, linking EV research closely to the etiology of GC.

We performed a literature search in PubMed, Web of Science, and Google Scholar for articles published from January 2010 to May 2026. The core search terms included “gastric cancer”, “exosomes”, “extracellular vesicles”, “tumor microenvironment”, “immune evasion”, “biomarker”, “targeted therapy”, and “engineered exosomes”. We prioritized original research articles and reviews published in English. For clinical translation studies, particular weight was given to studies with large cohort sizes, multicenter design, and external validation. Our search strategy aimed to capture the most impactful and recent contributions to the field while providing a balanced perspective on conflicting or unresolved questions.

## 2 Exosomes in the GC microenvironment

**FIGURE 1:**
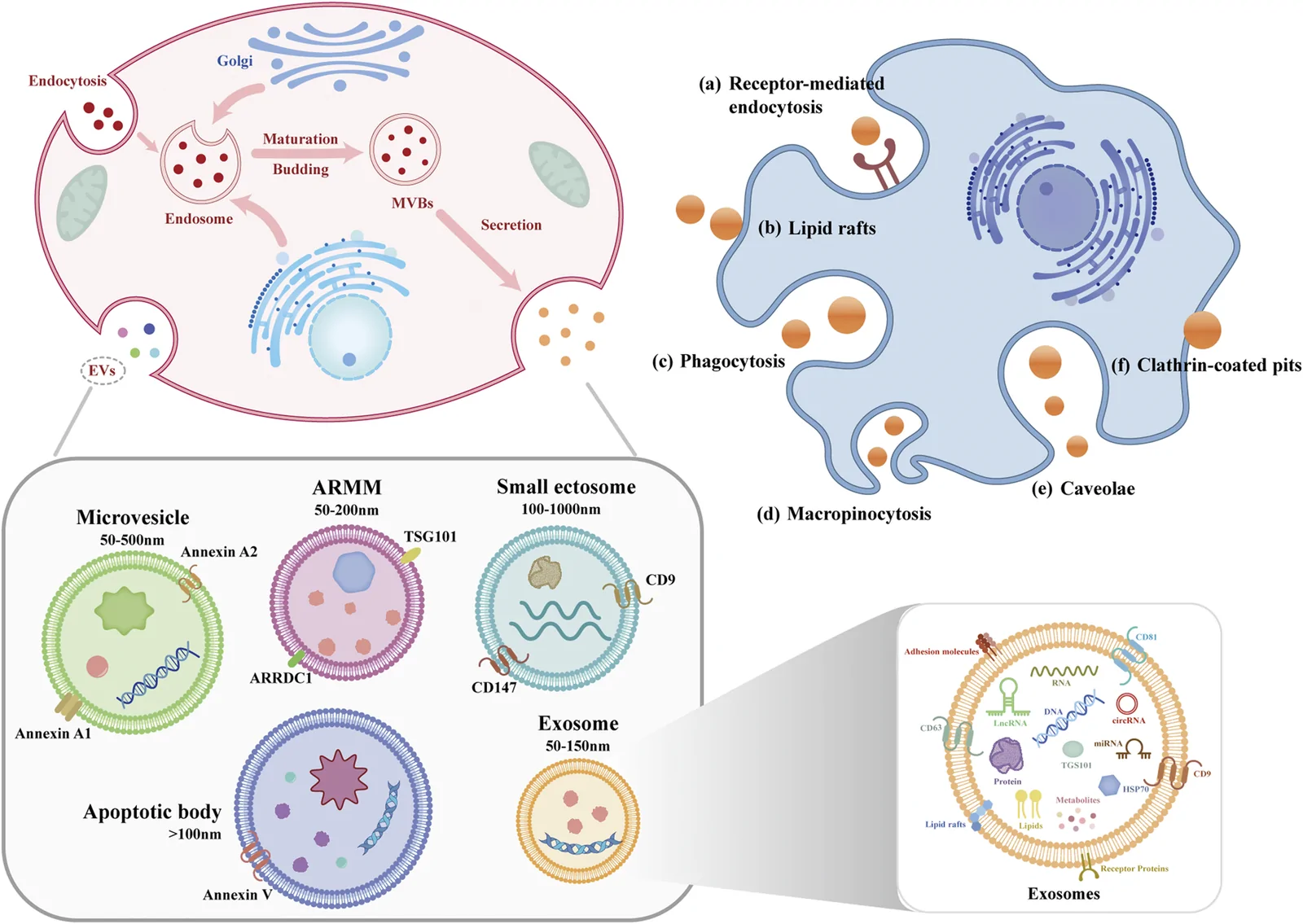
Overview of extracellular vesicles (EVs) biogenesis and subtypes.

Virtually all eukaryotic cells release nanoscale EVs rich in proteins, nucleic acids, and lipids, thereby facilitating intercellular molecular exchange (Lu et al., 2025; Jin et al., 2025). Based on their size and biogenesis pathways, EVs are classified into three major categories: small EVs (approximately 30–150 nm in diameter), which include exosomes and small ectosomes; medium EVs (approximately 200–800 nm), such as microvesicles; and large EVs (≥1,000 nm), encompassing apoptotic bodies and large vesicles (Tang L. et al., 2025). Exosomes represent the most thoroughly studied EV subtype. With a diameter of 30–150 nm, they exhibit a characteristic cup-shaped appearance under electron microscopy and express surface markers including CD9, CD63, CD81, Hsp70, and TSG101 (Zheng et al., 2025; Li X. et al., 2025; Liu H. et al., 2025). Exosome biogenesis initiates within the endosomal pathway and proceeds through a series of tightly regulated steps. Early endosomes arise from inward budding of the plasma membrane and, with additional input from the Golgi apparatus, mature into late endosomes. During this maturation, cargo is sorted into intraluminal vesicles, their accumulation converts the endosome into a multivesicular body (MVB) (Liu H. et al., 2025). MVBs are then directed either to lysosomes or autophagosomes for cargo degradation, or to the plasma membrane, where they release intraluminal vesicles as exosomes (Wang M. et al., 2018). Once secreted, exosomes interact with recipient cells through multiple mechanisms: ligand–receptor contacts can activate downstream signaling pathways; direct membrane fusion delivers luminal cargo into the cytoplasm; and endocytic uptake internalizes the entire vesicle (Tang L. et al., 2025). Cholesterol, ceramide and lipid raft constituents concentrate in the exosomal membrane, enhancing its stability and promoting target cell engagement through raft-dependent interactions (Jin et al., 2025) (Figure 1).

Exosomes vary markedly depending on their cell of origin. Their molecular cargo, including proteins, lipids, DNA, mRNA, miRNA, lncRNA, circRNA, and metabolites, closely reflects the identity and physiologic state of the parent cell (Pan et al., 2025) (Figure 1). In GC, tumor-derived exosomes actively contribute to the formation of a pre-metastatic niche, and the accumulation of ascitic fluid further primes the peritoneum for metastatic seeding. As key mediators, these exosomes not only exhibit organ-specific chemotaxis to precisely target the peritoneum but also facilitate tumor cell colonization by establishing an immunosuppressive microenvironment that removes natural barriers to peritoneal metastasis (Zhang et al., 2026).

## 3 Exosomes in the initiation and progression of GC

The initiation and progression of GC are not driven by tumor cells alone. As key messengers of intercellular communication, exosomes connect tumor cells, immune cells, and stromal cells into a complex regulatory network. This chapter comprehensively examines this network across six dimensions: proliferation, immunity, vasculature, stroma, metastasis, and drug resistance.

### 3.1 Exosomes drive malignant proliferation of GC cells

#### 3.1.1 *Helicobacter pylori* infection and the occurrence of GC

*Helicobacter pylori* infection is a major etiological factor in gastric carcinogenesis, with OMVs playing a central role in this process. Once internalized by neighboring or distant cells, these vesicles reshape the local immune milieu, sustain chronic inflammation, and drive the epithelium toward malignant transformation (Wang J. et al., 2025). OMVs shed by *H. pylori* are particularly rich in virulence-associated molecules, including CagA, VacA, urease, BabA/SabA, and the chaperonin GroEL. These OMVs engage host epithelial and immune cells through receptor interactions, endocytic uptake, or direct membrane fusion, thereby amplifying bacterial influence well beyond the immediate colonization site. Notably, OMVs can traverse the epithelial barrier and enter the vasculature of the lamina propria, suggesting systemic reach (Gratie et al., 2025).

OMVs are shed directly from the *H. pylori* outer membrane and represent a prokaryotic secretion system that delivers bacterial virulence factors into host cells. In contrast, the exosomes described in the following paragraphs are vesicles of host eukaryotic origin, released by H. pylori-infected gastric epithelial cells, immune cells, and stromal cells. These host-derived exosomes carry a molecular cargo that reflects the altered physiological state of the infected cell and operate downstream of the initial OMV-host interaction, sustaining and amplifying the pro-carcinogenic microenvironment over the long term. Thus, OMVs and host exosomes represent two sequential but mechanistically distinct vesicle systems in H. pylori-driven gastric carcinogenesis.

*H. pylori*-derived OMVs ferry CagA and VacA into host tissues, where they engage Toll-like receptors (TLRs) on macrophages and gastric epithelial cells. In response, macrophages secrete TNF-α, IL-6, and IL-1β, while epithelial cells release IL-8. In GC patients, exosomes of host origin recovered from gastric mucosal washings retain the capacity to provoke inflammation (Choi et al., 2017). GroEL, the bacterial Hsp60 ortholog, further amplifies this inflammatory cascade. Recognized by TLR4 and TLR2 on both epithelial and immune cells, GroEL activates NF-κB and MAPK signaling, driving the transcription of additional pro-inflammatory cytokines, and also activates the NLRP3 inflammasome (Gratie et al., 2025). Under the persistent oxidative and inflammatory stress of chronic infection, host Hsp60 can redistribute from mitochondria to the cytoplasm and plasma membrane and is packaged into exosomes via lipid raft-mediated internalization for secretion (Gratie et al., 2025).

*Helicobacter pylori* infection also remodels host cell functions through the exosomal pathway. Exosomes shed by H. pylori-infected GES-1 cells enhance the invasive and transendothelial migratory capacity of recipient cells *in vitro*, with HIF-1α among the EV-associated proteins potentially driving the emergence and progression of gastric precancerous lesions (González et al., 2022). A proteomic survey of exosomes from H. pylori-exposed host cells identified 120 proteins uniquely enriched in these vesicles, with HSP60 standing out as a key suppressor of apoptosis in infected cells (Li Y. et al., 2023). In a separate study, bone marrow mesenchymal stem cells challenged with *H. pylori* secreted exosomes that markedly accelerated proliferation, migration, and invasion *in vitro*, as well as tumor growth and metastasis *in vivo*, an effect attributed to a pronounced upregulation of thrombospondin-2 within the EV cargo (Qi et al., 2023). CagA, a major *H. pylori* virulence effector, shapes the immune landscape of GC. Wang and colleagues revealed that CagA elevates PD-L1 levels on tumor-derived exosomes by suppressing p53 and miRNA-34a, which in turn curbs CD8^+^ T cell expansion and facilitates immune escape (Wang et al., 2023a).

#### 3.1.2 Exosomes derived from GC and GC microenvironment cells exert pro-proliferative effects

**FIGURE 2:**
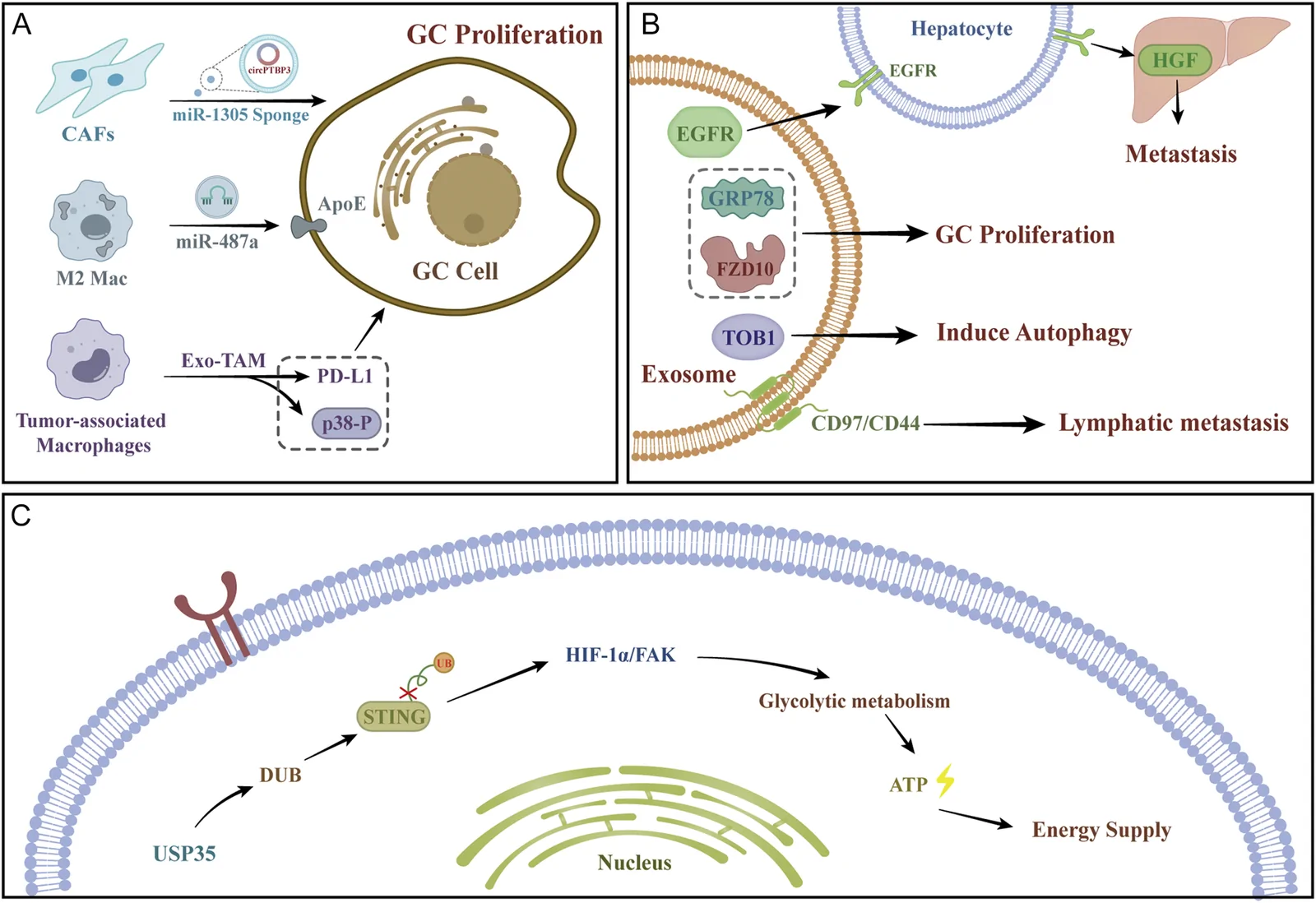
Exosome-driven proliferative signaling in gastric cancer. **(A)** Exosomes from CAFs, M2 macrophages and tumor-associated macrophages carry miR-1305 sponge, apolipoprotein E, miR-487a, phosphorylated p38 and PD-L1 to fuel GC cell proliferation. **(B)** GC cell-derived exosomes transfer EGFR to engage hepatocyte HGF signaling.It also carries GRP78, FZD10, TOB1 and CD97/CD44, which can sustain proliferation, induce autophagy and facilitate lymphatic metastasis. **(C)** USP35-mediated deubiquitination cooperates with upregulated HIF-1α/FAK and STING pathways, enhanced glycolytic metabolism and boosted ATP energy supply to drive drug resistance.

GC-derived exosomes were first shown to drive cell proliferation via the PI3K/Akt and MAPK/ERK axes (Qu et al., 2009). Subsequently, a wide range of exosomal cargoes from Cancer-associated fibroblasts (CAFs), TAMs, and GC cells have been implicated in promoting proliferation, predominantly through these same core pathways. CAF-derived exosomal circ_0088300 sponges miR-1305 (Shi H. et al., 2021). TAM-derived exosomes enhance p38 phosphorylation and PD-L1 expression (Wang et al., 2021b). M2 macrophage-derived exosomal miR-487a downregulates TIA1, and apolipoprotein E transferred by these same vesicles engages the PI3K-Akt axis to enhance invasiveness (Zheng et al., 2018). Additional exosomal cargoes, including miR-3184-5p, miR-519a-3p, circNRIP1, circITCH, and circNEK9, have also been associated with proliferative regulation (Tang L. et al., 2025; Qiu et al., 2022; Zhang et al., 2019; Wang et al., 2021c; Yu et al., 2021; Yue et al., 2021; Yang et al., 2021). These and other molecules are summarized in Table 1 (Figure 2A).

**TABLE 1:** The functional mechanisms of exosomes in the development of GC.

Molecule	Source	Pathway	Functional dimension	Ref.
circ_0088300	CAFs	miR-1305 sponge	Proliferation, migration, invasion	Shi et al. (2021a)
Unidentified	TAMs	p38, PD-L1	Proliferation, motility	Wang et al. (2021b)
miR-487a	M2 macrophages	TIA1	Proliferation	Zheng et al. (2018)
Apolipoprotein E	M2 macrophages	PI3K-Akt	Migration, invasion	Zheng et al. (2018)
miR-3184-5p	Serum (GC)	AKT, STAT3, IRE1	Apoptosis, growth suppression	Tang et al. (2025a)
miR-519a-3p	Serum (GC with liver metastasis)	DUSP2, MAPK/ERK	Angiogenesis, liver metastasis	Qiu et al. (2022)
circNRIP1	GC cells	-	Proliferation	Zhang et al. (2019)
circITCH	GC cells	-	Proliferation	Wang et al. (2021c)
circNEK9	GC cells	-	Proliferation	Yu et al. (2021), Yue et al. (2021), Yang et al. (2021)
rs/tsRNAs (S2, S7, S10)	Plasma exosomes (GC)	ErbB, Hippo pathways	Proliferation	Yang et al. (2025)
circMAN1A2	GC cells	SFPQ, CDK4	G1/S progression	Shen et al. (2025)
CD97	GC exosomes	Adhesion molecules	Lymphatic dissemination	Liu et al. (2016)
CD44	GC exosomes	YAP, CPT1A, fatty acid oxidation	Lymphatic metastasis	Wang et al. (2022a)
EGFR	GC exosomes	Hepatocyte growth factor signaling	Liver pre-metastatic niche	Zhang et al. (2017)
FZD10	GC exosomes	-	Proliferation	Scavo et al. (2019)
GRP78	GC exosomes	AKT	Proliferation, migration	Tsurusawa et al. (2022), Iha et al. (2022)
TOB1	GC exosomes	Autophagy	Cell survival	Wang et al. (2022b)
USP35	GC cells	STING, HIF-1α/FAK, glycolysis	Proliferation, adhesion	Yan et al. (2025)
PD-L1	GC exosomes	PD-1 on T cells, IL-6/STAT3 in MDSCs	T cell inhibition, MDSC expansion	Li et al. (2024a)
TGF-β	Tumor exosomes	T cells, Tregs	Treg differentiation, effector T cell suppression	Tankov et al. (2024), Marar et al. (2021)
LSD1	GC cells	PD-L1 loading	T cell suppression	Shen et al. (2022a)
miR-135b-5p	GC exosomes	Vγ9Vδ2 T cells	Impaired effector capacity	Li et al. (2022)
THBS1	GC exosomes	Vγ9Vδ2 T cells (m6A)	Enhanced cytotoxicity	Li et al. (2023a)
circMAN1A2	GC exosomes	SFPQ in CD8^+^ T cells	TCR signaling inhibition	Shen et al. (2025)
SERPINE1 (PAI-1)	GC exosomes	JAK2/STAT3, let-7g-5p, SOCS7	M2 polarization	Ye et al. (2025)
miR-519a-3p	GC exosomes	DUSP2, MAPK/ERK	M2 polarization	Qiu et al. (2022)
miR-92b-5p	GC exosomes	PLXNC1, STAT3	M2 polarization	Yi et al. (2024)
BGN	GC exosomes	NONO, CXCL10, CXCR3	M2 polarization, feedback loop	Li et al. (2025b)
circGLIS3	—	—	M2 polarization	Xiao et al. (2024)
ElNF1-AS1	—	—	M2 polarization	Liu et al. (2024)
miR-541-5p	—	—	M2 polarization	Zhang et al. (2024c)
HMGB1	GC exosomes	TLR4/NF-κB	M2 polarization, N2 polarization	Ma et al. (2023), Zhang et al. (2018b)
MALAT1	M2 TAM exosomes	δ-catenin, HIF-1α	Aerobic glycolysis	Wang et al. (2024)
Apolipoprotein E	M2 TAM exosomes	PI3K-Akt	Migration	Zheng et al. (2018)
miR-21	M2 TAM exosomes	PTEN, PI3K/AKT	Chemoresistance	Zheng et al. (2017)
circTEX2	M2 TAM exosomes	miR-145/ABCC1	Chemoresistance	Qu et al. (2024)
2,3-BdpM	L. salivarius bEVs	FPR1, MAPK, NF-κB	M1 polarization, antitumor immunity	Yu et al. (2026)
miR-107	GC exosomes	MDSCs	MDSC expansion	Ren et al. (2019)
miR-4745-5p/miR-3911	Neutrophil exosomes	GC cells	Metastasis	Zhang et al. (2024a)
miR-9-3p	N2 TAN exosomes	GC cells	Feedback regulation	Dong et al. (2025c)
TGF-β	GC exosomes	NK cells	Impaired cytotoxicity	Tang et al. (2024)
miR-552-5p	GC exosomes	NK cells	Impaired cytotoxicity	Tang et al. (2024)
GroEL	*H. pylori* OMVs	TLR4, TLR2	Th1/Th17 vs. Treg polarization	Gratie et al. (2025)
CagA	*H. pylori*	p53/miR-34a, PD-L1	CD8^+^ T cell suppression	Yong et al. (2025)
miR-130a	GC exosomes	C-MYB	Angiogenesis	Huang et al. (2019)
miR-23a	GC exosomes	PTEN	Angiogenesis	Du et al. (2020)
X26nt	GC exosomes	VE-cadherin	Vascular permeability	Chen et al. (2021)
circSHKBP1	GC exosomes	VEGF secretion	Angiogenesis	Xie et al. (2020)
circFCHO2	GC exosomes	miR-194-5p, JAK1/STAT3	Angiogenesis	Zhang et al. (2022)
miR-29a/c	GC exosomes	VEGF (suppression)	Anti-angiogenesis	Zhang et al. (2016)
YB-1	GC exosomes	VEGF, Ang-1, MMP-9, IL-8	Angiogenesis	Wang et al. (2023b), Xue et al. (2020)
GRP78	GC exosomes	AKT	Endothelial proliferation/migration	Iha et al. (2022)
MSC-derived exosomes	MSCs	—	Angiogenic control	Akad et al. (2023)
miR-519a-3p	GC exosomes (intrahepatic macrophages)	M2 polarization	Pre-metastatic niche, angiogenesis	Qiu et al. (2022)
VWF	GC exosome-endothelium interaction	Integrin αvβ3	Exosome adhesion, vascular permeability	Wang et al. (2025a)
TGF-β	GC exosomes	SMAD pathway	Fibroblast-to-CAF conversion	Jin et al. (2025)
miR-146a	GC exosomes	NF-κB pathway	Fibroblast-to-CAF conversion	Jin et al. (2025)
miR-10b-5p	GC exosomes	PTEN, TGF-β pathway	Proliferation, fibroblast-to-CAF conversion, pericyte conversion	Yan et al. (2021)
TGF-β/Smad	GC exosomes	MSCs	MSC-to-CAF differentiation	Gu et al. (2012)
Wnt5a	Lymph node-metastatic GC exosomes	YAP signaling, bone marrow MSCs	MSC reprogramming	Wang et al. (2021a)
UBR2	p53-deficient BM-MSC exosomes	Wnt/β-catenin	GC progression	Mao et al. (2017)
miR-199a-5p	CAF exosomes	EMT	EMT induction	Jin et al. (2025)
MMPs	CAF exosomes	ECM	ECM degradation	Jin et al. (2025)
CD9-positive exosomes	CAFs	—	Scirrhous GC migration	Miki et al. (2018)
PKM2	GC exosomes	NF-κB in fibroblasts	Immune suppression	Fan et al. (2019)
miR-139	CAF exosomes	MMP11	Tumor inhibition	Xu et al. (2019)
LTBP1	PDPN^+^LTBP1^+^ CAF exosomes	TGF-β signaling in HSCs	Liver pre-metastatic niche	Zhao et al. (2025b)
Menin	CAF exosomes	HSPA6/JNK/JunD, EMT	Proliferation, invasion, lung metastasis	Wang et al. (2025c)
Unidentified	GC exosomes	NFAT1/c-Rel, STAT5	FOXP3^+^ fibroblast reprogramming	Kimura et al. (2025)
FERMT2	GC-CAFs	ZEB2, miR-138/miR-200a	CAF activation	He et al. (2025a)
COL6A1	GC exosomes (FERMT2-driven)	TGF-β autocrine loop	CAF reactivation	He et al. (2025a)
HIF1A-AS3	Hypoxic MSC exosomes	miR-142-3p/miR-24-3p, PROX1, WNT	MSC-to-CAF conversion	Xu et al. (2025)
Cortactin	Dysplastic cell exosomes	RAB27A, actin network	Exosome secretion, malignant transformation	Guenther et al. (2025)
Integrin αvβ3	GC exosomes	Periostin, FAK/Pyk2	Peritoneal homing, pre-metastatic niche	Hoshino et al. (2015)
circ_0000437	GC exosomes	HSPA2/ERK	Lymphatic metastasis	Shen et al. (2022b)
lncRNA ZFAS1	GC tissues, serum exosomes	—	Metastatic spread	Pan et al. (2017)
miR-1246	Plasma sEVs (H. pylori-positive GC)	Lymphatic endothelial cells	Lymphangiogenesis, vascular remodeling	Lu et al. (2024)
CD44	GC exosomes	YAP, CPT1A, fatty acid oxidation	Lymph node metastasis	Wang et al. (2022a)
CD97	GC exosomes	CD55, CD44v6, α5β1, CD31	Lymphatic metastasis	Liu et al. (2016)
NPR1	GC cells	PKG, hormone-sensitive lipase, fatty acid β-oxidation	Lymph node metastasis	Fu et al. (2025)
miR-21-5p	GC exosomes	TGF-β/Smad	MMT, peritoneal metastasis	Li et al. (2018b)
miR-106a	GC exosomes	Smad7	Peritoneal metastasis	Zhu et al. (2022), 2020
miR-15b-3p	GC exosomes	Apoptotic regulators	Peritoneal metastasis	Zhu et al. (2022)
miR-486-5p	GC exosomes (depleted)	EMT	Peritoneal metastasis	Lin et al. (2021)
NNMT	GC exosomes	TGF-β/Smad2	Peritoneal metastasis	Zhu et al. (2021)
METTL3/miR-17–92 cluster	GC exosomes	RAB27A, SRCIN1, SRC	Exosome biogenesis, peritoneal macrophage reprogramming	Li et al. (2025a)
circPTBP3	GC exosomes	TFAP2B, SGK1	MMT, peritoneal metastasis	Dong et al. (2025b)
USP35	GC exosomes	STING, HIF-1α/FAK, MMT	Peritoneal metastasis	Yan et al. (2025)
TGF-β1/FERMT2/COL6A1 loop	GCAFs, GC exosomes	TGF-β/SMAD, anoikis resistance	Peritoneal colonization	He et al. (2025a)
miR-21	Peritoneal lavage exosomes	—	Peritoneal recurrence marker	Tokuhisa et al. (2015)
miR-1225-5p	Peritoneal lavage exosomes	—	Peritoneal recurrence marker	Tokuhisa et al. (2015)
miR-29b-3p	Peritoneal lavage exosomes	—	Peritoneal metastasis prognosis	Ohzawa et al. (2020)
ARHGEF12 E620K/ITGA6	GC exosomes	Rap1, ovarian fibroblasts	Ovarian metastasis	Zhang et al. (2025b)
VWF	GC exosome-endothelium interaction	Integrin αvβ3, ADAMTS-13	Hematogenous metastasis, vascular permeability	Wang et al. (2025a)
LTBP1	PDPN^+^LTBP1^+^ CAF exosomes	TGF-β, hepatic stellate cells, CCL11/CCR3	Hepatic pre-metastatic niche	Zhao et al. (2025b)
miR-769-5p	GC exosomes	Caspase-9, p53	Cisplatin resistance	Jing et al. (2022)
miR-500a-3p	GC exosomes	FBXW7	Cisplatin resistance, stemness	Lin et al. (2020)
circPVT1	GC exosomes	miR-30a-5p/YAP1	Cisplatin resistance	Shi et al. (2021b), Yao et al. (2021)
lnc00852	GC exosomes	miR-514a-5p/COMMD7	Cisplatin resistance	Kumata et al. (2018), Cao et al. (2023)
miR-21	M2 TAM exosomes	PTEN, PI3K/AKT	Cisplatin resistance	Zheng et al. (2017)
miR-3681-3p	M2 TAM exosomes	MLH1	Cisplatin resistance	Wei et al. (2025)
circTEX2	M2 TAM exosomes	miR-145/ABCC1	Cisplatin resistance	Qu et al. (2024)
c-Met siRNA (engineered)	Exosomes	c-Met	Cisplatin sensitization	Zhang et al. (2020b)
Anti-miR-214	Exosomes	—	Cisplatin sensitization	Wang et al. (2018c)
miR-106a-5p/miR-421	GC exosomes	AP2e hypermethylation	5-FU resistance	Sun et al. (2019)
Unidentified	MSC exosomes	Ca^2+^/Raf/MAPK/ERK	5-FU resistance	Lin et al. (2022), Ji et al. (2015)
miR-155-5p	Paclitaxel-resistant GC exosomes	EMT	Paclitaxel resistance	Wang et al. (2018b)
DACT3-AS1	CAF exosomes (loss of)	—	Oxaliplatin resistance	Qu et al. (2023)
miR-522	CAF exosomes	ALOX15, ferroptosis	Acquired chemoresistance	Zhang et al. (2020a)
circUBR5	GC exosomes	miR-1208/CYP19A1, ACAT1/PSMD14	Cisplatin resistance, cholesterol metabolism	Jiang et al. (2026)
HIF1A-AS3	Hypoxic MSC exosomes	miR-142-3p/miR-24-3p, PROX1	Oxaliplatin resistance	Xu et al. (2025)
miR-9-3p	N2 TAN exosomes	ACSL4, ferroptosis	Oxaliplatin resistance	Dong et al. (2025c)
CCT6A	CAF exosomes	β-catenin, c-Myc, DDIT4, TXNIP	Cisplatin resistance, glycolysis	Sun et al. (2025)
PD-L1	Tumor exosomes	PD-1 on T cells	Immunotherapy resistance	Wu et al. (2023), Poggio et al. (2019)
miR-21-5p	M2 TAM exosomes	METTL3, CD70, Tregs	Anti-PD-1 resistance	Ning et al. (2023)
NESC (engineered)	NK cell exosomes	CLDN4, ROS	Radiosensitization	Dong et al. (2025a)

Recently discovered rsRNAs and tsRNAs within exosomes have expanded the regulatory repertoire of exosomal non-coding RNAs. Yang et al. showed that elevated levels of rs/tsRNAs (S2, S7, and S10) in plasma exosomes from GC patients promote malignant proliferation by directly suppressing key molecules in the ErbB pathway, including CDKN1A, PAK3, and NRG4, and in the Hippo pathway, including NF2, TEAD3, LATS1, and SMAD4 (Yang et al., 2025).

However, most of these findings derive from established cell lines or xenograft models. Whether the same mechanisms hold in spontaneous gastric tumors where the microenvironment co-evolves with the disease over years has not been tested. In addition, given that cargoes from H. pylori-derived OMVs, tumor cell-derived exosomes, CAF-derived exosomes, and macrophage-derived exosomes all converge on PI3K/Akt and MAPK/ERK, it remains unclear whether individual molecules reported in single studies carry genuine specificity or rather reflect shared cellular stress responses amplified by *in vitro* culture.

Beyond the classic miRNA sponge mechanism, circMAN1A2 drives G1/S progression in GC cells by tethering and stabilizing the RNA-binding protein SFPQ (Shen et al., 2025). SFPQ subsequently upregulates CDK4 expression and promotes retinoblastoma protein phosphorylation, thereby accelerating cell cycle progression. Silencing either circMAN1A2 or SFPQ results in G1 phase arrest and reduced proliferative capacity. This finding reveals a mechanism by which circRNAs influence the cell cycle through modulating protein stability.

At the protein level, multiple exosome-carried proteins have been implicated in proliferation and metastatic priming. Exosomal CD97 and CD44 drive lymphatic dissemination (Shen X. et al., 2022; Wang M. et al., 2022). Exosomal EGFR inserts into the plasma membrane of hepatocytes and primes the liver for metastatic colonization (Zhang et al., 2017). FZD10 sustains GC cell proliferation (Scavo et al., 2019). GRP78 promotes GC cell proliferation and migration (Tsurusawa et al., 2022; Iha et al., 2022), whereas TOB1 affects cell survival by inducing autophagy (Wang Y. et al., 2022). These protein cargoes are also listed in Table 1 (Figure 2B).

Studies described above indicate that exosome-mediated pro-proliferative effects exhibit a high degree of molecular redundancy. Whether derived from H. pylori-infected host cells, tumor cells, or CAFs and macrophages, exosomes drive proliferation by converging on PI3K/Akt and MAPK/ERK. This pattern explains why targeting tumor cells alone often yields limited efficacy, and suggests that interfering with exosome biogenesis or their common downstream pathways may hold greater therapeutic promise than targeting individual cargo molecules.

Malignant proliferation is also modulated by metabolic reprogramming. Yan et al. found that USP35 stabilizes STING through deubiquitination, thereby activating the HIF-1α/FAK pathway and promoting glycolytic metabolism, which provides energy support for GC cell adhesion and proliferation (Yan et al., 2025). RNA-seq analysis confirmed that USP35 overexpression upregulates glycolysis-related genes. This finding reveals a mechanism by which deubiquitinating enzymes influence tumor cell behavior through metabolic regulation (Figure 2C).

However, several caveats merit attention. First, most of the aforementioned findings are derived from established cell lines or xenograft models, which may not fully recapitulate the complex, evolved tumor microenvironment of spontaneous gastric carcinogenesis. Second, exosomal cargoes from diverse cellular sources, including tumor cells, CAFs, and macrophages, all funnel into PI3K/Akt and MAPK/ERK, it remains unclear whether the individual molecules reported in single studies carry genuine functional specificity or merely reflect shared cellular stress responses amplified by *in vitro* culture. Notably, the convergence of *H. pylori* OMV-derived virulence factors onto these same pathways operates through distinct upstream receptors and signaling adaptors, representing a parallel but mechanistically separate route to pathway activation. Third, the field has largely focused on “which molecule” and “which pathway,” but less on “contribution of signaling” and “dynamics during disease progression”. Future studies should prioritize in functional inactivation models using conditional knockout of key exosome biogenesis regulators in specific cell types, combined with single-cell resolution tracking, to dissect the causative versus permissive roles of exosomal signaling in proliferation. Additionally, leveraging patient-derived organoids and orthotopic models will be critical to validate whether these mechanisms operate in the human disease context.

### 3.2 Exosomes remodel the GC microenvironment to support GC cells growth

#### 3.2.1 Immunoregulation

Tumor cells harness exosomes to escape immune detection and reshape the surrounding immune landscape. GC cell-derived exosomes directly carry PD-L1, which engages PD-1 on T cells to inhibit T cell activation and proliferation while simultaneously fueling MDSC expansion via IL-6/STAT3 signaling (Li H. et al., 2024). Tumor-derived exosomes also deliver TGF-β to drive Treg differentiation and suppress effector T cell activity (Tankov et al., 2024; Marar et al., 2021). LSD1 promotes PD-L1 enrichment within GC exosomes (Shen D.-D. et al., 2022). GC-derived exosomal miRNAs further modulate T cell behavior, with miR-135b-5p undermining the effector capacity of Vγ9Vδ2 T cells (Li et al., 2022), and THBS1, via m6A modification, augmenting their cytotoxic potential (Li J. et al., 2023).

Shen and colleagues recently identified circMAN1A2 as an exosomal effector that suppresses CD8^+^ T cell immunity in GC (Shen et al., 2025). This circRNA, packaged via hnRNPA2B1, is internalized by T cells and binds the RNA-binding protein SFPQ, shielding it from FBXW11-driven K48-linked ubiquitination and proteasomal degradation. The accumulated SFPQ inhibits phosphorylation of TCR signaling components, including LCK, ZAP70, PLCγ1, and LAT, thereby curtailing IFN-γ and TNF-α production. *In vivo*, administration of the TCR agonist LYP-IN-3 reversed the accelerated tumor growth induced by circMAN1A2 overexpression. This study provides evidence that an exosomal circRNA exerts dual functions by regulating the stability of the same target protein, promoting G1/S transition in tumor cells while inhibiting antitumor immunity in T cells.

Tumor-associated macrophages (TAMs) are profoundly influenced by exosomes. GC cells drive macrophage M2 polarization through several exosome-mediated routes. Ye and colleagues identified SERPINE1 as a key mediator that promotes M2 polarization via a dual pathway (Ye et al., 2025). SERPINE1 engages the JAK2/STAT3 axis to boost let-7g-5p transcription, resulting in elevated let-7g-5p loading into GC-derived exosomes. Once internalized by macrophages, exosomal let-7g-5p suppresses SOCS7, relieving its inhibition on STAT3 phosphorylation and driving M2 polarization. Silencing SERPINE1 markedly suppressed tumor growth and curbed M2-type TAM infiltration *in vivo*. Beyond SERPINE1, several other exosomal cargoes have been shown to induce M2-like polarization, including miR-519a-3p, miR-92b-5p, BGN, circGLIS3, ElNF1-AS1, miR-541-5p, and HMGB1, although independent validation for most of these factors remains limited (Xiao et al., 2024; Liu et al., 2024; Zhang Y. et al., 2024; Ma et al., 2023; Qiu et al., 2022; Yi et al., 2024; Li W. et al., 2025). Tumor-derived exosomes can also educate monocytes to become pro-tumor TAMs expressing PD-1, which adopt an M2-like phenotype and inhibit CD8^+^ T cell activity (Wang F. et al., 2018).

Macrophages are not merely passive recipients. Exosomes shed by M2-polarized TAMs re-engage tumor cells and the stroma, reinforcing a self-amplifying circuit. MALAT1 drives aerobic glycolysis in GC cells via δ-catenin and HIF-1α (Wang et al., 2024). Apolipoprotein E enhances migration via PI3K-Akt (Zheng et al., 2018). miR-21 and circTEX2 promote chemoresistance through distinct mechanisms (Zheng et al., 2017; Qu et al., 2024).

Li et al. recently uncovered a positive feedback loop initiated by the exosomal proteoglycan BGN (Li W. et al., 2025). Upon uptake by macrophages, BGN binds NONO, driving M2 polarization while simultaneously upregulating CXCL10 secretion. Macrophage-derived CXCL10 engages CXCR3 on GC cells, triggering JAK/STAT1 signaling and fueling proliferation, EMT, and metastatic spread *in vivo*. This study reveals a “tumor-macrophage-tumor” circuit mediated by a single exosomal protein.

Beyond exosomes from tumor and stromal compartments, intratumoral bacteria-derived vesicles are emerging as participants in shaping the GC immune microenvironment. Yu et al. found that the gastric commensal bacterium Ligilactobacillus salivarius is enriched in GC patients who respond to immune checkpoint inhibitor therapy (Yu et al., 2026). L. salivarius-derived bacterial extracellular vesicles (bEVs) are selectively taken up by TAMs. The effector protein 2,3-diphosphoglycerate-dependent phosphoglycerate mutase (2,3-BdpM) carried by bEVs engages formyl peptide receptor 1 (FPR1) on macrophages, triggering MAPK and NF-κB signaling that steers the cells toward a pro-inflammatory M1-like state. The resulting M1-like macrophages boost granzyme B and IFN-γ output from CD8^+^ T cells. In multiple mouse models of GC, oral administration of L. salivarius or intraperitoneal injection of its bEVs significantly potentiated the antitumor efficacy of anti-PD-1 antibodies and even reversed resistance to immunotherapy (Yu et al., 2026). This finding reveals a mechanism by which intratumoral microbes regulate antitumor immunity across biological kingdoms through their secreted vesicles.

MDSCs and neutrophils are also targets of exosome-mediated immunosuppression. GC cell-derived exosomes promote MDSC expansion via miR-107 (Ren et al., 2019), and exosomal PD-L1 facilitates MDSC accumulation through IL-6/STAT3 (Li H. et al., 2024). GC cell-derived exosomes drive neutrophil polarization toward a pro-tumor N2-like phenotype via the HMGB1/TLR4/NF-κB pathway (Zhang X. et al., 2018) and upregulate PD-L1 expression through STAT3 (Shi et al., 2020b). Neutrophil-derived exosomes can transmit miR-4745-5p and miR-3911 to promote metastasis (Zhang J. et al., 2024). Dong and colleagues further traced a feedback loop in which GC-derived exosomal HMGB1 triggers NF-κB signaling in neutrophils, driving miR-9-3p expression. The resulting N2-polarized TANs release exosomes loaded with miR-9-3p, which are internalized by GC cells (Dong X. et al., 2025).

**FIGURE 3:**
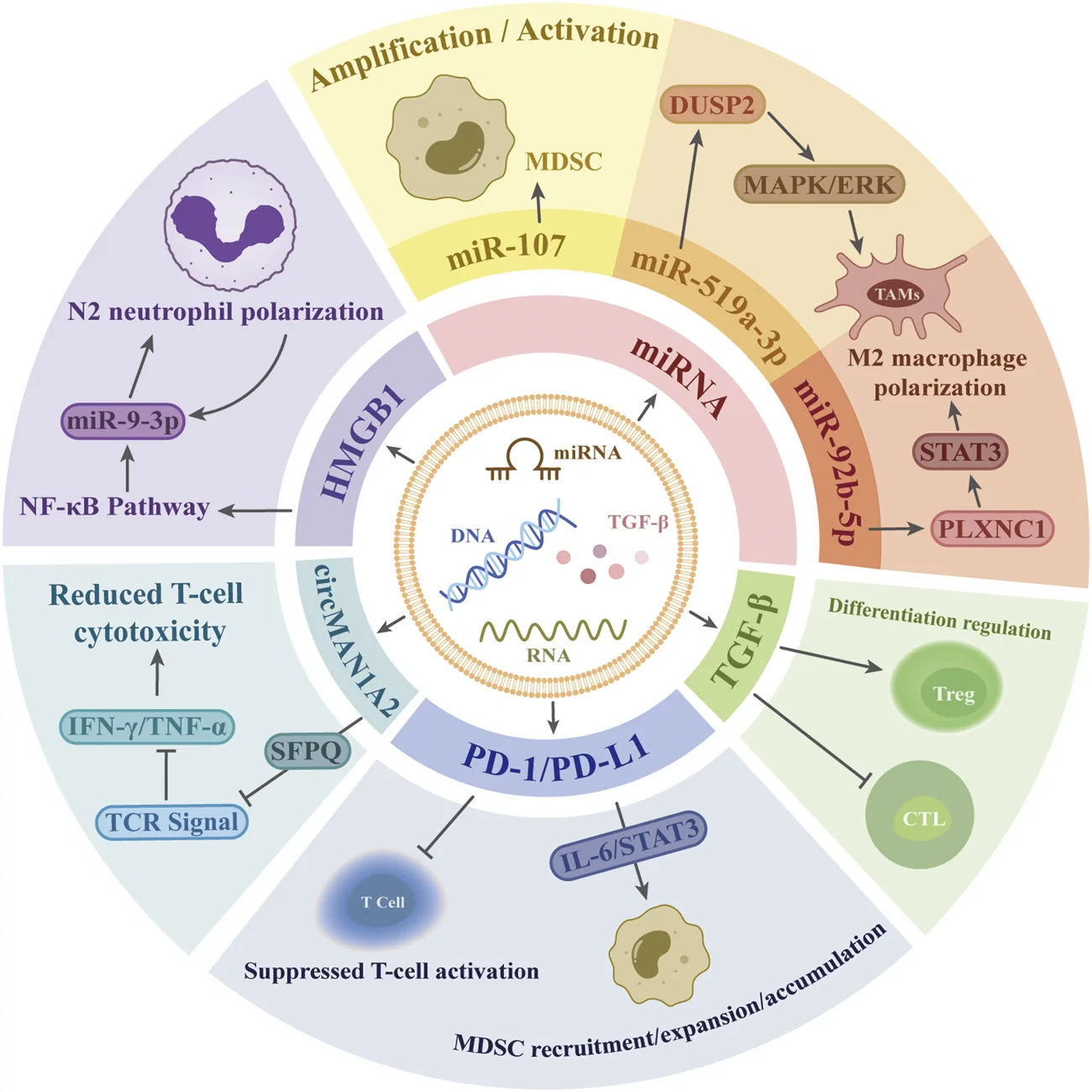
Exosome-mediated immune regulation in the gastric cancer microenvironment. Tumor-derived exosomes carry PD-L1, TGF-β, miRNAs and circRNAs to suppress T cell function and promote regulatory T cell differentiation. Exosomal PD-L1 activates IL-6/STAT3 signaling to expand MDSCs. Tumor exosomes also drive macrophage M2 polarization via molecules such as SERPINE, miR-519a-3p, miR-92b-5p and BGN. M2 macrophage exosomes further enhance tumor glycolysis, migration and chemoresistance. Exosomal HMGB1 induces N2 neutrophil polarization via NF-κB and miR-9-3p. Cross-talk also involves dendritic cells, NK cells, and bacterial Exosomes that reprogram macrophages toward an M1-like state.

Exosomes also modulate dendritic cell (DC) maturation and NK cell function. EBV-associated GC exosomes block DC maturation (Hinata et al., 2020), whereas heat-treated malignant ascites exosomes promote DC-driven CTL responses (Zhong et al., 2011). GC-derived exosomes impair NK cytotoxicity via TGF-β and miR-552-5p (Tang et al., 2024), while NK-derived exosomes can directly induce tumor cell apoptosis (Jin et al., 2025) (Figure 3).

In the context of GC etiology, *H. pylori* contributes to immune evasion through both OMV-dependent and host exosome-dependent mechanisms. *H. pylori*-derived OMVs deliver GroEL, whose structural resemblance to human Hsp60 enables molecular mimicry. Repeated exposure to GroEL-containing OMVs may lead to cross-reactivity during antigen processing, polarizing T-cell responses along two opposing pathways. The host can mount a pro-inflammatory Th1/Th17 response, while persistent high-dose exposure may drive the outgrowth of tolerogenic dendritic cells and regulatory T cells, culminating in local immunosuppression (Gratie et al., 2025). Additionally, *H. pylori* CagA upregulates exosomal PD-L1 by inhibiting the p53/miR-34a axis, suppressing CD8^+^ T cell function (Yong et al., 2025).

Collectively, exosome-mediated immune regulation exhibits a dynamic duality. Tumor-derived exosomes comprehensively construct an immunosuppressive network centered on T-cell dysfunction, with M2-type macrophages and N2-type neutrophils serving as accomplices. Bacterial extracellular vesicles (bEVs) derived from intratumoral probiotics such as L. salivarius can break this network by activating macrophages and rejuvenating antitumor immunity. This unity of opposites provides a strategic framework for immunotherapy: one approach is to inhibit the release or function of pathogenic tumor-derived exosomes, and another is to use engineered probiotics or bEVs as adjuvants to synergize with immune checkpoint inhibitors and reshape cold tumors into hot ones. Molecules such as circMAN1A2 exert opposing effects in tumor cells and T cells by regulating the same target protein SFPQ, revealing a molecular coupling between exosome-mediated immune evasion and tumor proliferation.

Despite these insights, the immunoregulatory field faces substantial challenges in translation. First, contradictory data exist for several cargoes. For instance, miR-21 delivered by M2-TAM exosomes promotes cisplatin resistance through PTEN suppression (Zheng et al., 2017), yet tumor-suppressive functions of the same miRNA have been documented in other GC settings. Differences in exosome isolation methods, macrophage polarization protocols, and the target gene repertoire of recipient cells likely contribute to these discrepancies, but systematic side-by-side comparisons are lacking. Second, the majority of immunomodulatory studies rely on *in vitro* co-culture systems that do not account for the spatiotemporal dynamics of immune cell trafficking *in vivo*. The relative contribution of exosomal PD-L1 versus surface PD-L1 on tumor cells to systemic T-cell exhaustion remains unresolved. Third, while the identification of pro-tumorigenic bacterial EVs is conceptually exciting, their *in vivo* stability, biodistribution, and potential off-target effects require thorough evaluation before therapeutic translation. Future work should employ multiplexed single-cell profiling of the TME following exosome perturbation, coupled with *in vivo* imaging of exosome trafficking, to map the cellular targets and temporal windows of exosomal immune modulation. Clinically, prospective cohort studies correlating circulating exosomal cargo profiles with dynamic changes in the systemic immune landscape during immunotherapy are urgently needed.

#### 3.2.2 Angiogenesis

GC cell-derived exosomes package an array of pro-angiogenic factors, including both non-coding RNAs and proteins. At the non-coding RNA level, miR-130a and miR-23a promote angiogenesis by targeting C-MYB and PTEN, respectively (Huang et al., 2019; Du et al., 2020). A specific 26-nt RNA (X26nt) reduces vascular endothelial cadherin expression and enhances vascular permeability (Chen et al., 2021). Among circRNAs, circSHKBP1 induces VEGF secretion (Xie et al., 2020) and circFCHO2 engages the JAK1/STAT3 axis by sequestering miR-194-5p (Zhang et al., 2022). Additional exosomal miRNAs and circRNAs with pro-angiogenic activity have been reported, although most await independent validation; these molecules are summarized in Table 1. Notably, exosomal miRNAs can exert bidirectional effects. miR-29a/c suppress angiogenesis by reducing VEGF expression, suggesting a delicate balancing mechanism (Zhang et al., 2016).

At the protein level, Y-box binding protein 1 (YB-1) upregulates VEGF, Ang-1, MMP-9, and IL-8 in vascular endothelial cells (Wang et al., 2023b; Xue et al., 2020). GRP78 drives endothelial cell proliferation and migration via AKT signaling (Iha et al., 2022). Mesenchymal stem cell-derived exosomes likewise contribute to angiogenic control (Akad et al., 2023). The hypoxic tumor microenvironment further drives exosome production and alters their pro-angiogenic cargo. Preclinical studies have confirmed that the VEGF inhibitor apatinib effectively blocks the pro-angiogenic effect of exosomes from irradiated GC cells on endothelial cells (Li G. et al., 2018).

Exosome-driven angiogenesis often precedes overt tumor expansion and helps prime the pre-metastatic niche. Exosomal miR-519a-3p, taken up by intrahepatic macrophages, steers them toward an M2-like state and creates a favorable microenvironment for liver metastasis by inducing angiogenesis (Qiu et al., 2022).

Increased vascular permeability is a prerequisite for metastasis. Recent studies have shown that von Willebrand factor (VWF) plays a critical role in the endothelial permeability induced by GC cell-derived exosomes. Highly adhesive VWF promotes the adhesion of these exosomes to the endothelium, facilitating disruption of the endothelial barrier (Wang C. et al., 2025).

Collectively, exosomes provide critical vascular support for local progression and distant metastasis of GC by inducing angiogenesis and increasing vascular permeability.

#### 3.2.3 Stromal remodeling and CAF activation

CAFs are among the most critical cellular components of the gastric stromal microenvironment. Exosomes mediate both the conversion of normal cells into CAFs and the feedback regulation that CAFs exert on tumor cells.

**FIGURE 4:**
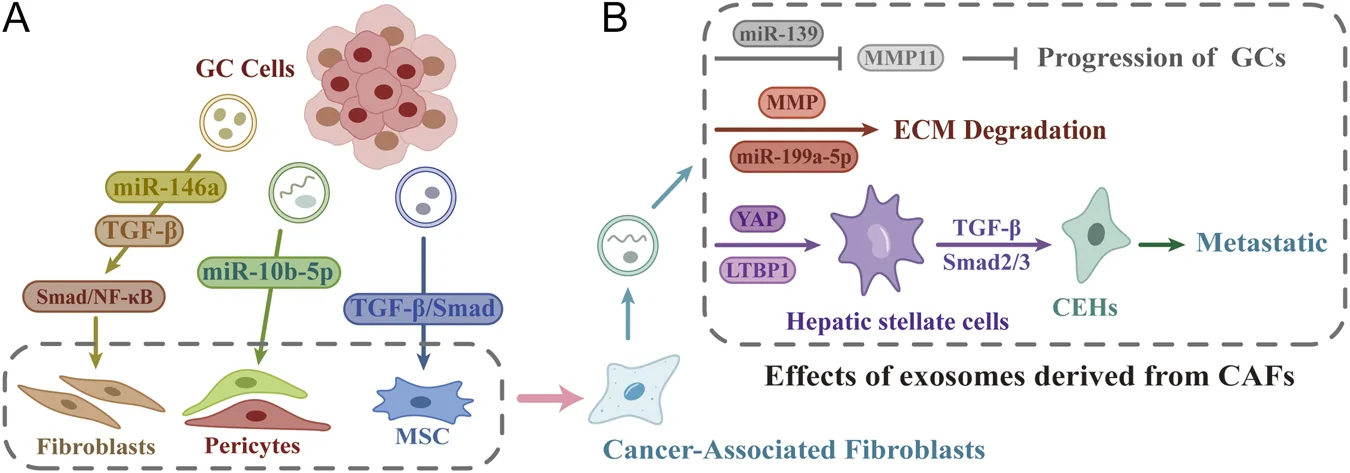
Stromal remodeling and CAF activation in gastric cancer. **(A)** Tumor cell-derived exosomes educate normal stromal cells. Exosomal miR-10b-5p and TGF-β trigger SMAD signaling, while exosomal miR-146a and PKM2 activate NF-κB, driving the conversion of fibroblasts, pericytes and mesenchymal stem cells into CAFs. **(B)** CAF-derived exosomes reshape the microenvironment locally and distantly. miR-139 suppresses MMP11, and MMPs degrade ECM. CAF exosomal LTBP1 reaches the liver and activates TGF-β/Smad2/3 in hepatic stellate cells, cooperating with cancer-educated hepatocytes to establish a pre-metastatic microenvironment.

During CAF formation, tumor cell-derived exosomes act as educators. GC cell-derived exosomes deliver TGF-β and miR-146a to activate SMAD or NF-κB pathways, inducing the conversion of normal fibroblasts into CAFs (Jin et al., 2025). Exosomal miR-10b-5p, elevated in serum of advanced GC patients, enhances proliferation by targeting PTEN and directly converts fibroblasts into CAFs through TGF-β signaling (Yan et al., 2021). GC cell-derived exosomes also induce differentiation of mesenchymal stem cells into CAFs via TGF-β/Smad (Gu et al., 2012) and trigger pericyte conversion through miR-10b-5p (Yan et al., 2021) (Figure 4A). Additional exosomal cargoes involved in CAF formation, including Wnt5a from lymph node-metastatic GC cells and UBR2 from p53-deficient bone marrow MSC exosomes, are listed in Table 1 (Wang M. et al., 2021; Mao et al., 2017).

Once established, CAFs release exosomes that reinforce the malignant behavior of tumors. CAF exosomal miR-199a-5p triggers EMT, matrix metalloproteinases break down extracellular matrix barriers, and signaling proteins activate pro-growth cascades including Wnt/β-catenin (Jin et al., 2025). CD9-positive exosomes from CAFs enhance the migratory capacity of scirrhous GC cells (Miki et al., 2018). GC cells release PKM2-laden exosomes that activate NF-κB in fibroblasts (Fan et al., 2019). CAF-derived exosomes do not always exert pro-tumor effects. Exosomal miR-139 released by CAFs inhibits tumor cell growth and motility through MMP11 suppression (Xu et al., 2019). CAFs are not a homogeneous population. Using single-cell sequencing, Zhao and colleagues identified a PDPN^+^LTBP1^+^ CAF subset that highly expresses pro-tumorigenic factors, with LTBP1 regulated by YAP signaling. These CAFs release exosomes that reach the liver, where they activate TGF-β signaling in hepatic stellate cells through LTBP1, remotely remodeling the metastatic niche (Zhao Z. et al., 2025) (Figure 4B).

Malignant transformation of gastric mucosal epithelial cells is a multi-stage process in which dysplasia represents the final precancerous stage. Guenther et al. revealed the core role of cortactin in the transition from dysplasia to invasive carcinoma (Guenther et al., 2025). Cortactin is highly expressed in gastric dysplasia and carcinoma tissues but low in normal mucosa. In dysplasia-derived organoids, cortactin depletion did not affect proliferation but significantly reduced malignant morphological features. Mechanistically, cortactin cooperates with RAB27A to stabilize the branched actin network at the apical membrane, facilitating multivesicular body tethering to the plasma membrane and promoting exosome discharge. Cortactin-null dysplastic cells were unable to attract PDGFRα^+^ fibroblasts or F4/80^+^ macrophages *in vivo* and failed to form adenocarcinomas or solid tumors, with only small cystic structures persisting. These findings anchor exosome function at the earliest stage of gastric carcinogenesis, demonstrating that dysplastic cell-derived exosomes serve as key messengers in constructing a pro-tumor microenvironment.

CAF exosomes also transfer Menin into GC cells, where it activates the HSPA6/JNK/JunD axis to drive EMT and promote lung metastasis (Wang S.-H. et al., 2025). Kimura et al. reported the existence of a fibroblast subpopulation expressing FOXP3 in the GC microenvironment, predominantly in stage III/IV patients, with reduced CD8^+^ T cell infiltration in their vicinity (Kimura et al., 2025). GC cell-derived exosomes induce FOXP3 expression in fibroblasts, and FOXP3^+^ fibroblasts promote peritoneal metastasis through CDH11-mediated Wnt signaling.

He et al. uncovered a bidirectional positive feedback loop between tumor cells and GC-CAFs (He C. et al., 2025). FERMT2 is highly expressed in GC-CAFs and upregulates ZEB2 through competitive binding of miR-138 and miR-200a, which transcriptionally activates α-SMA to maintain the myofibroblast phenotype. Activated GC-CAFs secrete TGF-β1, which upregulates FERMT2 in GC cells via SMAD2/3. FERMT2 in turn promotes secretion of COL6A1-carrying exosomes by tumor cells. Once taken up by GC-CAFs, these exosomes reinforce the activated state through a TGF-β autocrine loop, forming a closed circuit. Hypoxia within the tumor microenvironment further drives stromal reprogramming. Xu et al. discovered that hypoxia activates lncRNA HIF1A-AS3 in MSCs via HIF-1α, which sponges miR-142-3p and miR-24-3p to relieve suppression of PROX1, ultimately driving MSC conversion into α-SMA^+^/FAP^+^ CAFs through WNT signaling (Xu et al., 2025).

Together, the above mechanisms reveal an exosome-mediated bidirectional positive feedback loop between tumor cells and CAFs that drives the malignant progression of GC.

However, the stromal compartment remains underexplored relative to tumor cell-autonomous mechanisms. A critical caveat is the heterogeneity of CAFs. While single-cell sequencing has identified subsets such as PDPN^+^LTBP1^+^ CAFs with distinct pro-metastatic functions, most functional studies, including the FERMT2/COL6A1 feedback loop and HIF1A-AS3-driven conversion described above, still pool CAFs as a bulk population, obscuring subset-specific contributions and potential antagonism between CAF subtypes. Furthermore, the temporal sequence of CAF activation during gastric carcinogenesis, from chronic gastritis to dysplasia to invasive carcinoma, is poorly defined in exosome-mediated stromal remodeling. The study by Guenther et al. on cortactin in dysplastic organoids represents a rare glimpse into the precancerous stage, but systematic investigations across the entire carcinogenic spectrum are lacking. Methodologically, the field must move beyond TGF-β as the universal driver of CAF conversion and explore other signaling axes that may operate in a context-dependent manner. Future research should integrate lineage-tracing models with exosome reporter systems to track the origin, fate, and functional plasticity of CAF subpopulations educated by tumor-derived exosomes. From a therapeutic perspective, disrupting the tumor-CAF exosomal crosstalk, rather than depleting CAFs globally, may offer a more precise strategy to reverse stromal desmoplasia without compromising tissue repair functions.

### 3.3 Exosomes drive GC invasion and metastasis

Exosomes shed by GC cells fuel local invasion and distant spread. They prime target tissues for metastatic seeding by reshaping the microenvironment, triggering EMT, and breaching the mesothelial barrier. Surface integrins on exosomes help dictate organ-specific homing. Integrin αvβ3 recognizes the RGD motif of periostin within the peritoneal matrix, activating FAK/Pyk2 signaling and boosting cancer cell adhesion and motility. This event also recruits macrophages and fosters an immunosuppressive pre-metastatic niche (Hoshino et al., 2015). Exosomes can also push tumor cells into an invasive state by triggering EMT. In addition to circMAN1A2 modulating T cell function through SFPQ, Menin in CAF-derived exosomes directly induces EMT in GC cells by activating the HSPA6/JNK/JunD pathway and promotes lung metastasis (Wang S.-H. et al., 2025).

#### 3.3.1 Lymphatic metastasis

**FIGURE 5:**
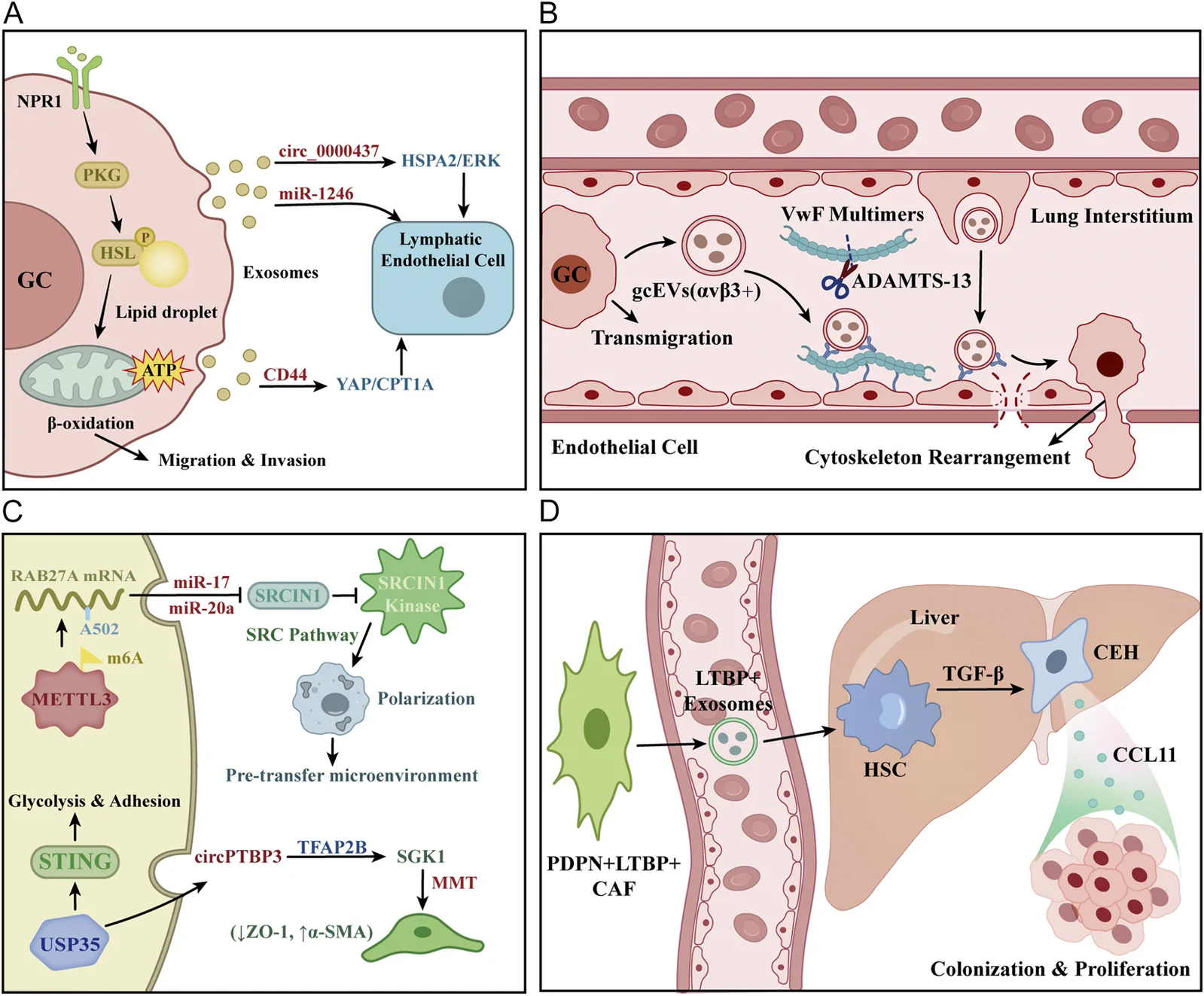
Related mechanisms of metastasis in GC. **(A)** Lymphatic metastasis: GC promotes mitochondrial β-oxidation via the NPR1-PKG-HSL axis to enhance migration and invasion; exosomes carrying various factors act on lymphatic endothelial cells, facilitating lymph node metastasis. **(B)** Hematogenous metastasis: GC-derived gcEVs promote tumor hematogenous spread through transmembrane migration and modulation of vascular permeability. **(C)** Peritoneal metastasis: METTL3 upregulates RAB27A via m6A modification, while miR-17/20a inhibits SRCIN1 to regulate SRC signaling; USP35 activates STING to promote glycolysis and adhesion; the circPTBP3/TFAP2B axis activates SGK1 and induces MMT, collectively driving peritoneal metastasis. **(D)** Hepatic colonization and proliferation: PDPN^+^LTBP^+^ CAF-derived exosomes activate HSCs to secrete CCL11, which promotes GC liver colonization and proliferation via the CCL11/receptor axis.

Lymphatic spread dominates GC dissemination. Exosomes from GC cells carry non-coding RNAs that remodel the lymphatic niche. Among these, circ_0000437 has been shown to promote lymphangiogenesis via HSPA2/ERK (Shen X. et al., 2022), and miR-1246 stimulates lymphangiogenesis specifically in H. pylori-positive GC patients (Lu et al., 2024). LncRNA ZFAS1 has also been implicated, although its mechanism in lymphatic remodeling is less well characterized (Pan et al., 2017) (Figure 5A). Exosomal CD44 reprograms fatty acid oxidation through YAP and CPT1A (Wang M. et al., 2022), and CD97 involves synergistic action of CD55, CD44v6, α5β1, and CD31 (Liu et al., 2016). Lymph node spread marks a turning point in GC. Fu and colleagues identified NPR1 as a key driver of lymph node metastasis (Fu et al., 2025). NPR1 protein was more abundant in lymphovascular emboli and nodal metastases than in matched primary tumors, and elevated NPR1 correlated with shorter overall survival. NPR1 engages PKG to phosphorylate hormone-sensitive lipase, liberating free fatty acids from lipid droplets. These fatty acids fuel mitochondrial β-oxidation, enhancing ATP production to power cell migration and invasion. Knocking down NPR1 or blocking fatty acid oxidation reduced migration and invasion *in vitro* and curtailed nodal metastasis *in vivo*. RGD-tagged exosome mimetics loaded with NPR1 siRNA homed to tumors and suppressed lymph node spread in animal models (Fu et al., 2025). These and other molecules implicated in lymphatic metastasis—several of which have been validated in multiple models, whereas others remain single-study observations—are summarized in Table 1.

#### 3.3.2 Peritoneal metastasis

Peritoneal spread drives much of the lethality observed in advanced GC. GC-derived exosomes pave the way for tumor cell seeding by disrupting the mesothelial barrier and triggering mesothelial-to-mesenchymal transition (MMT) (Deng et al., 2017; Li Q. et al., 2018). Several miRNAs orchestrate this process. miR-21-5p is one of the most consistently reported mediators, activating the TGF-β/Smad axis to promote MMT (Li Q. et al., 2018). miR-106a modulates Smad7 (Zhu et al., 2022; Zhu et al., 2020), miR-15b-3p enhances migration through suppression of apoptotic regulators (Zhu et al., 2022), and miR-486-5p is depleted in pro-metastatic GC exosomes, a loss that favors EMT (Lin et al., 2021). NNMT drives peritoneal metastasis through TGF-β/Smad2 (Zhu et al., 2021).

Epitranscriptomics is beginning to intersect with exosome biology in peritoneal metastasis. Li and colleagues discovered that METTL3 is upregulated in GC cells, where it installs m6A marks at the A502 site of RAB27A mRNA, lifting its translation and ramping up exosome output (Li S. et al., 2025) (Figure 5C). METTL3 also promotes packaging of the miR-17-92 cluster into exosomes. These vesicles are taken up by peritoneal macrophages, in which miR-17 and miR-20a silence SRCIN1. Loss of SRCIN1 unleashes SRC kinase activity, driving macrophages toward an immunosuppressive state with increased IL-10 and TGF-β and reduced IL-1, IL-6, and TNF-α. The re-educated macrophages suppress CD8^+^ T cell proliferation and cytotoxicity, establishing an immunosuppressive pre-metastatic niche in the peritoneal cavity. In immunocompetent mice, exosomes from METTL3-high GC cells induced peritoneal niche formation and accelerated metastasis, and depletion of macrophages largely abolished this effect. This work delineates a complete pathway from m6A-driven exosome biogenesis to remote reprogramming of the peritoneal immune landscape.

A pivotal early event in peritoneal metastasis is MMT of peritoneal mesothelial cells. Dong and colleagues demonstrated that GC-derived exosomes drive MMT, with loss of ZO-1 and increased expression of α-SMA, FSP1, collagen I, and fibronectin (Dong C. et al., 2025) (Figure 5C). The key mediator is exosomal circPTBP3. After entering mesothelial cells, it travels to the nucleus, recruits TFAP2B to the SGK1 promoter, and upregulates SGK1 transcription, which promotes MMT. In patients, plasma exosomal circPTBP3 levels were markedly higher in those with peritoneal metastasis (AUC = 0.866) and correlated with deeper invasion, nodal involvement, and advanced TNM stage, although this finding was derived from a single retrospective cohort and requires prospective validation. High circPTBP3 also predicted shorter survival. Silencing circPTBP3 in exosomes reduced peritoneal fibrosis and tumor implantation *in vivo*.

USP35, a deubiquitinating enzyme overexpressed in peritoneal nodules, also contributes to peritoneal metastasis through two complementary mechanisms (Yan et al., 2025). Within GC cells, USP35 stabilizes STING and activates HIF-1α/FAK signaling, shifting metabolism toward glycolysis and enhancing adhesion to peritoneal mesothelial cells. Concurrently, exosomal USP35 is taken up by mesothelial cells and induces MMT. *In vivo*, knockdown of USP35 reduced peritoneal nodule formation, whereas exosomal USP35 promoted dissemination.

He et al. uncovered a positive feedback loop involving TGF-β1, FERMT2, and COL6A1 that drives peritoneal colonization (He C. et al., 2025). TGF-β1 from GCAFs arms GC cells against anoikis, and tumor-derived exosomal COL6A1 reactivates GCAFs. Blocking FERMT2 or applying the TGF-β receptor inhibitor SB-431542 markedly reduced peritoneal tumor burden in mice.

Exosomal cargo has also been explored as a source of biomarkers for peritoneal spread. miR-21 and miR-1225-5p in peritoneal lavage exosomes may flag postoperative peritoneal recurrence (Tokuhisa et al., 2015). Low miR-29b-3p levels identify patients at higher risk for peritoneal metastasis and poorer survival (Ohzawa et al., 2020). These molecules are listed in Table 1.

The above mechanisms reveal the dual roles of exosomes in peritoneal metastasis. They home to target organs via integrin-mediated tropism and clear obstacles for tumor cell colonization by inducing MMT and remodeling the immune microenvironment. The involvement of m6A modification and deubiquitinating enzymes links exosome biology to upstream molecular regulatory networks, offering new therapeutic targets for intervention in peritoneal metastasis.

Ovarian metastasis (Krukenberg tumor) represents a special form of peritoneal metastasis. Through whole-exome sequencing, Zhang et al. identified the ARHGEF12 E620K gain-of-function mutation as a specific driver event in GC ovarian metastasis (Zhang M. et al., 2025). This mutation activates Rap1 signaling, leading to upregulation of integrin ITGA6. GC cells harboring the E620K mutation secrete exosomes enriched with ITGA6, which are preferentially taken up by ovarian fibroblasts, particularly the estrogen receptor-positive subpopulation, inducing their conversion into CAFs and establishing a pre-metastatic niche. *In vivo*, GC cells expressing the E620K mutation spontaneously form ovarian metastases, whereas ARHGEF12 knockdown markedly suppresses metastasis.

#### 3.3.3 Hematogenous metastasis

Hematogenous metastasis represents a major route for distant spread of GC, with the lung being one of the most common target organs. Wang et al. revealed a critical role for von Willebrand factor (VWF) in the hematogenous metastasis of GC (Wang C. et al., 2025). GC cell-derived exosomes activate endothelial cells, prompting the release of hyperadhesive VWF multimers. These multimers bind to integrin αvβ3 on the surface of GC-derived exosomes, mediating their adhesion to the vascular endothelium under blood flow shear stress. The anchored exosomes are internalized by endothelial cells, leading to cytoskeletal rearrangement, disruption of tight junctions, and increased vascular permeability. Circulating GC cells then undergo transendothelial migration and enter the lung interstitium to form metastatic colonies. ADAMTS-13, a specific VWF-cleaving protease, reduces exosome adhesion to the endothelium by cleaving hyperadhesive VWF multimers. *In vivo*, administration of recombinant human ADAMTS-13 significantly reduced the number of metastatic nodules in the lungs of mice (Figure 5B).

#### 3.3.4 Hepatic metastasis

The liver is a common target organ for distant metastasis of GC. Zhao et al. identified a specialized subpopulation of PDPN^+^LTBP1^+^ CAFs within primary tumors that remotely induce the formation of a pre-metastatic niche in the liver through exosomes (Zhao Z. et al., 2025). Activation of YAP signaling in PDPN^+^LTBP1^+^ CAFs upregulates LTBP1 expression, which is packaged into exosomes. These LTBP1-rich exosomes travel to the liver, where they are taken up by hepatic stellate cells and activate TGF-β signaling, converting them into CAF-educated HSCs. The activated HSCs secrete CCL11, which recruits GC cells expressing CCR3 via the CCL11/CCR3 axis, promoting their colonization and proliferation. LTBP1 expression is significantly higher in liver metastases than in peritoneal or ovarian metastases, and the abundance of PDPN^+^LTBP1^+^ CAFs correlates negatively with overall survival. Silencing LTBP1 in PDPN^+^ CAFs markedly reduced the number of liver metastatic nodules (Figure 5D).

In summary, exosome-driven metastasis depends on two key steps. Surface integrins such as αvβ3 guide exosomes to specific organs. Exosomal cargoes, including non-coding RNAs and proteins, remodel local stromal cells into a supportive environment. This reprogrammed niche in turn recruits circulating tumor cells. Blocking exosome homing by neutralizing integrins or disrupting niche formation could be a viable strategy to prevent distant metastasis in gastric cancer.

Nevertheless, the metastasis field confronts the formidable translational hurdles. First, organotropism studies have predominantly focused on integrin profiles, but the molecular logic governing exosome uptake by specific resident cells in each metastatic site remains opaque. For instance, how LTBP1^+^ CAF exosomes are selectively taken up by hepatic stellate cells rather than other liver-resident populations is not understood. Second, the transition from micrometastasis to macrometastasis is rarely modeled in exosome studies, as most experimental endpoints are measured within weeks, missing the protracted dormancy and reactivation phases that characterize clinical metastasis. Third, while peritoneal metastasis is clinically the most devastating pattern in GC, the current mechanistic understanding is largely derived from intraperitoneal injection models that bypass the natural dissemination route. Whether exosomes shed from primary tumors indeed reach the peritoneal cavity in sufficient quantities and maintain functional integrity in ascites fluid needs quantitative pharmacokinetic assessment. Future directions should include: (i) developing organ-on-a-chip or microfluidic systems to model multi-step metastasis under hemodynamic flow; (ii) employing barcoded exosome libraries to screen for organ-specific homing codes in a high-throughput manner; (iii) designing therapeutic exosome-based drugs that competitively block integrin-ligand interactions and prevent pre-metastatic niche formation, and (iv) conducting longitudinal liquid biopsy studies to track exosomal signatures as early warning indicators of impending metastatic relapse.

### 3.4 Exosome-mediated treatment resistance

Tumor cells can acquire drug resistance through exosome-mediated mechanisms. Exosomes shed by drug-tolerant cells transfer resistant traits to previously sensitive cells.

#### 3.4.1 Chemotherapy resistance

**FIGURE 6:**
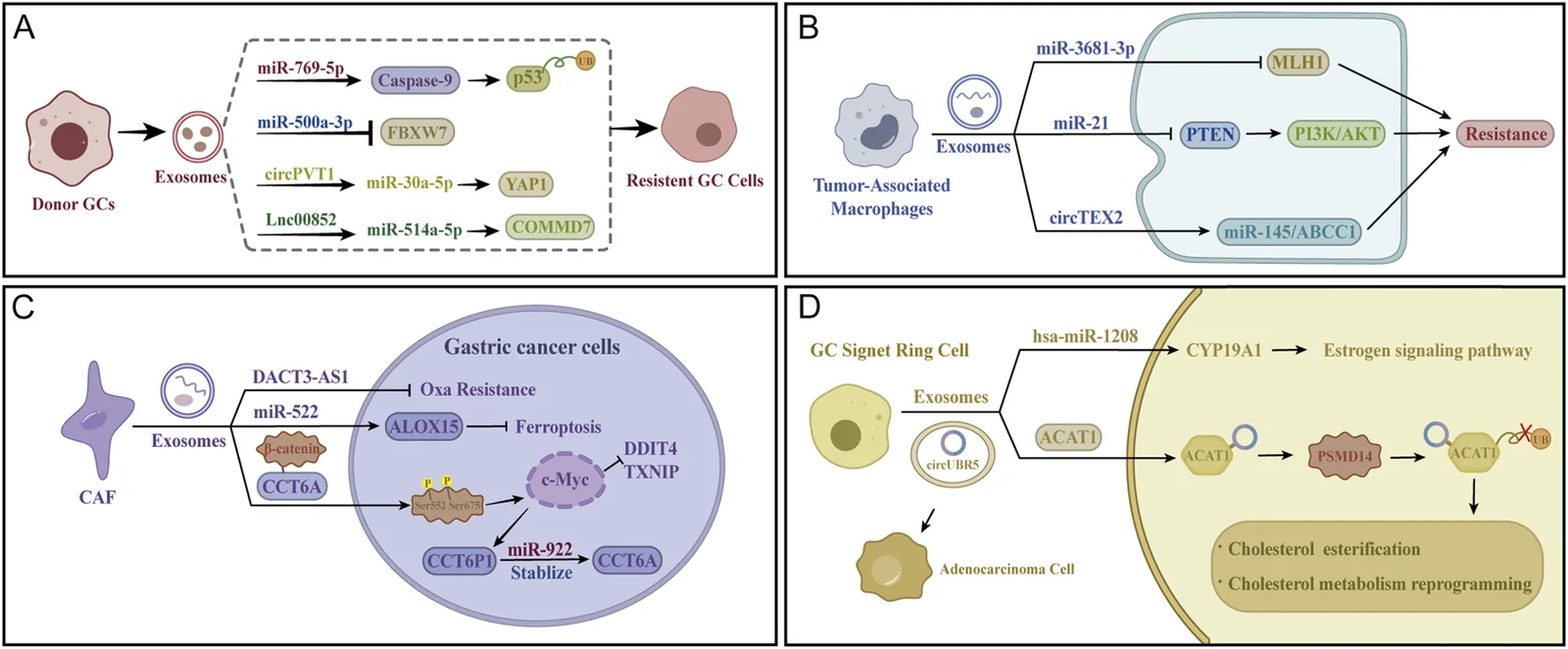
Exosomal mechanisms of chemotherapy resistance in gastric cancer. **(A)** Cisplatin-resistant gastric cancer cells secrete exosomes carrying miR-769-5p that targets caspase-9 and drives p53 ubiquitination, miR-500a-3p that suppresses FBXW7, circPVT1 modulating YAP1, and lnc00852 regulating COMMD7 via miR-514a-5p. **(B)** Tumor-associated macrophages deliver exosomal miR-21 to silence PTEN and activate PI3K/AKT, exosomal miR-3681-3p to suppress MLH1, and circTEX2 to enhance resistance through the miR-145/ABCC1 axis. **(C)** CAF-derived exosomes drive oxaliplatin resistance through DACT3-AS1 loss, miR-522 targeting ALOX15 to block ferroptosis, and CCT6A promoting β-catenin phosphorylation at Ser552/Ser675 to activate c-Myc, which represses DDIT4 and TXNIP. **(D)** Exosomal circUBR5 from gastric signet ring cells propagates cisplatin resistance to adenocarcinoma cells by sponging hsa-miR-1208 to upregulate CYP19A1 and estrogen signaling, and by scaffolding ACAT1 with PSMD14 to stabilize ACAT1 and reprogram cholesterol metabolism via enhanced esterification.

For cisplatin, GC-derived exosomes spread resistance via a variety of non-coding RNAs. Among these, circPVT1 has been independently reported by two groups to modulate the miR-30a-5p/YAP1 axis (Shi J. et al., 2021; Yao et al., 2021), providing a degree of cross-validation. miR-769-5p targets caspase-9 and drives p53 degradation (Jing et al., 2022). miR-500a-3p suppresses FBXW7, enhancing drug tolerance and stemness (Lin et al., 2020), and lnc00852 regulates COMMD7 through miR-514a-5p (Kumata et al., 2018; Cao et al., 2023) (Figure 6A). Stromal exosomes also shape cisplatin responses. M2-polarized TAMs dispatch exosomal miR-21, which silences PTEN and activates PI3K/AKT (Zheng et al., 2017). These macrophages also deliver miR-3681-3p to suppress MLH1 (Wei et al., 2025) and circTEX2, which engages the miR-145/ABCC1 axis (Qu et al., 2024) (Figure 6B). Additional exosomal cargoes implicated in cisplatin resistance are listed in Table 1.

Conversely, exosomes can tilt the balance toward drug sensitivity. Delivery of c-Met siRNA via exosomes renders GC cells more vulnerable to cisplatin (Zhang Q. et al., 2020), and exo-anti-214 alleviates cisplatin resistance (Wang X. et al., 2018). In the context of 5-fluorouracil, AP2e hypermethylation fuels resistance via exosomal transfer of miR-106a-5p and miR-421 (Sun et al., 2019), and MSC-derived exosomes activate the Ca^2+^/Raf/MAPK/ERK cascade (Lin et al., 2022; Ji et al., 2015). For paclitaxel, resistant GC cells release exosomal miR-155-5p, driving sensitive cells into EMT and resistance (Wang M. et al., 2018). For oxaliplatin, CAF-derived exosomal DACT3-AS1 normally restrains resistance and its loss unleashes drug tolerance (Qu et al., 2023), while CAF-secreted miR-522 blocks ferroptosis by targeting ALOX15 (Zhang H. et al., 2020) (Figure 6C).

Cisplatin resistance remains a central barrier to durable responses in GC. Jiang and colleagues recently dissected how circUBR5 enforces this resistance in gastric signet ring cell carcinoma (Jiang et al., 2026). circUBR5 works through two separate routes. It sponges hsa-miR-1208, liberating CYP19A1 and activating estrogen signaling. It also acts as a protein scaffold, binding the cholesterol-esterifying enzyme ACAT1 and recruiting the deubiquitinase PSMD14. This removes ubiquitin from ACAT1, stabilizes the enzyme, and drives cholesterol esterification along with lipid droplet buildup. circUBR5 is exported via tumor exosomes and propagates the resistant phenotype across the tumor microenvironment (Jiang et al., 2026) (Figure 6D).

The hypoxia-induced transformation of MSCs into CAFs also drives chemotherapy resistance. GC cells treated with conditioned medium from hypoxia-exposed MSCs exhibited a 2- to 3-fold increase in the IC50 for oxaliplatin and a 50%–60% reduction in apoptosis. Knockdown of HIF1A-AS3 or overexpression of miR-142-3p/miR-24-3p reversed this resistant phenotype (Xu et al., 2025).

Ferroptosis, an iron-dependent form of cell death driven by lipid peroxidation, has emerged as a critical mechanism underlying chemotherapy resistance. Dong et al. reported that N2-TANs suppress ferroptosis in GC cells via exosomal miR-9-3p, which directly targets the 3′UTR of ACSL4 mRNA and inhibits its expression (Dong X. et al., 2025). The miR-9-3p-mediated downregulation of ACSL4 significantly reduces the sensitivity of GC cells to ferroptosis inducers and oxaliplatin. *In vivo*, treatment with N2-Ex reversed the antitumor effect of oxaliplatin, whereas knockdown of miR-9-3p or overexpression of ACSL4 restored drug sensitivity.

Sun et al. uncovered a mechanism by which CAF-derived exosomal CCT6A drives chemotherapy resistance (Sun et al., 2025). CCT6A binds β-catenin, promotes its phosphorylation at Ser552/Ser675 and nuclear translocation, and enhances c-Myc transcriptional activity. c-Myc then transcriptionally represses DDIT4 and TXNIP, relieving the inhibition of glycolysis and enhancing both stemness and cisplatin resistance. Knockdown of CCT6A or treatment with the β-catenin inhibitor XAV939 or the c-Myc inhibitor 10058-F4 significantly reversed drug resistance. A positive feedback loop was also identified in which c-Myc transcriptionally activates both CCT6A and its pseudogene CCT6P1. CCT6P1 acts as a ceRNA that sponges miR-922 and stabilizes CCT6A, forming a continuously amplified pro-resistance signal.

#### 3.4.2 Immunotherapy resistance

Exosomes also attenuate responses to anti-PD-1/PD-L1 immunotherapy. Tumor-derived vesicles carry functional PD-L1 that cripples T cell activity systemically, and strategies that reduce exosomal PD-L1 output amplify the impact of checkpoint blockade (Wu et al., 2023; Poggio et al., 2019; Sun et al., 2024). M2-polarized macrophages dispatch exosomal miR-21-5p, which downregulates METTL3 in cancer cells and allows CD70 expression to rise. The subsequent increase in Tregs and terminally exhausted T cells erodes anti-PD-1 efficacy. In animal models, the anti-CD70 antibody cusatuzumab restored therapeutic sensitivity (Ning et al., 2023).

#### 3.4.3 Radiotherapy resistance

Radiotherapy is frequently employed for locally advanced gastric cancer, but its efficacy is often limited by intrinsic or acquired radioresistance. Dong et al. engineered a CLDN4-targeting exosome platform termed NESC by conjugating a high-affinity CLDN4-binding peptide and a membrane-curvature sensing domain onto NK cell-derived small extracellular vesicles (Dong A. et al., 2025). NESC selectively bound to CLDN4-overexpressing GC cells and elevated intracellular ROS levels. When combined with irradiation, NESC potentiated DNA double-strand break formation. In patient-derived GC organoids and organoid-derived xenograft models, NESC combined with radiotherapy markedly suppressed tumor growth and prolonged survival, effectively reversing the radioresistant phenotype.

Together, these findings indicate that exosomes mediate therapeutic resistance through similar mechanisms in both chemotherapy and immunotherapy, suggesting their potential as targets for combination therapy.

## 4 Exosomes as biomarkers for GC diagnosis

As a novel tool for liquid biopsy, exosomes have demonstrated substantial potential in the early detection, progression monitoring, and prognostic evaluation of GC. Their unique advantages stem from several biological characteristics. First, exosomes are widely distributed in body fluids such as blood, gastric juice, and ascites, enabling non-invasive sampling. Second, their lipid bilayer shields their cargo from degradation, offering greater detection stability compared to cell-free molecules in plasma. Third, the molecular composition of exosomes faithfully reflects the pathological status of the parent tumor cells (Zheng et al., 2025). In the development of liquid biopsy markers, cell-free miRNAs and exosomal miRNAs exhibit distinct advantages: the former provide high sensitivity due to their abundant presence, whereas the latter offer high specificity as they originate from tumor cells. Through a multicenter large-cohort study, Sui et al. systematically validated this concept and proposed a complementary strategy in which “cell-free miRNAs provide sensitivity, and exosomal miRNAs provide specificity” (Sui et al., 2025). Using small RNA sequencing and the XGBoost machine learning algorithm, the researchers screened and validated a diagnostic panel (Destinex) comprising five cell-free miRNAs and five exosomal miRNAs from 809 specimens. In the validation cohort, this panel achieved an area under the curve (AUC) of 94.8%, a sensitivity of 88.5%, and a specificity of 89.0% for distinguishing GC from healthy controls. Of particular importance, its AUC for pT1-stage early GC reached 96.8%, significantly outperforming traditional serum markers. Despite its promise, the Destinex panel requires prospective validation in real-world screening populations, as its performance was assessed in a retrospective cohort with predefined case-control ratios that may not reflect the lower prevalence of GC in general screening settings. The generalizability of this 10-marker signature to diverse ethnic populations also remains untested. Beyond RNA and proteins, DNA carried by exosomes also harbors rich diagnostic information. Among these, DNA methylation, as the most stable epigenetic modification, offers unique advantages for early cancer detection. Lin et al. recently developed a novel whole-genome methylation sequencing technique termed TEMPT, which successfully enables high-quality methylome library construction from sub-nanogram quantities of EV-DNA through a strategy combining single-adapter Tn5 fragmentation, enzymatic conversion, and terminal tailing (Lin et al., 2025). Applying this technique to analyze plasma EV-DNA from 58 patients with GC and gastric polyps, the study identified 647 differentially methylated regions. A random forest model built on these regions achieved an AUC of 0.8 in the validation cohort for distinguishing GC from benign lesions. However, this study had a limited sample size of only 58 patients, and the validation cohort was not entirely independent of the discovery set, raising the risk of overfitting. Given the small cohort, these findings should be considered exploratory. Whether the identified methylation markers can distinguish early-stage GC from high-risk premalignant conditions such as intestinal metaplasia or low-grade dysplasia requires further investigation.

### 4.1 Exosomal non-coding RNAs

Exosomal miRNAs have been evaluated as diagnostic and prognostic tools in GC. Serum exosomal miR-1246 decreases in early-stage disease and may rise with regression (Shi et al., 2020a). miR-23b helps predict recurrence and prognosis (Kumata et al., 2018), while miR-92a-3p has been proposed for early detection (Lu et al., 2021). For peritoneal spread, miR-29 and miR-181 correlate with metastatic risk and recurrence (Ohzawa et al., 2020; Yun et al., 2019), and miR-21 and miR-1225-5p in peritoneal washings may signal postoperative peritoneal metastasis (Tokuhisa et al., 2015).

Exosomal lncRNAs have also shown diagnostic promise. Plasma UEGC1 is elevated in early-stage GC and outperforms CEA (Lin et al., 2018). Circulating lncRNA-GC1 tracks disease progression more effectively than conventional markers (Guo et al., 2020), and serum HOTTIP may aid both diagnosis and prognosis (Wang M. et al., 2018; Zhao et al., 2018).In a large multicenter study, Cai and colleagues developed a four-lncRNA serum exosomal panel, termed the cd-score, comprising RP11.443C10.1, CTD-2339L15.3, LINC00567, and DGCR9. This panel achieved an AUC of 0.959 in the training cohort and 0.949 in external validation, substantially outperforming CEA, CA19-9, and CA72-4 (Cai et al., 2025). Although this panel showed excellent performance in the training and external validation cohorts, both cohorts were drawn from the same Chinese population, and its applicability to patients with non-Asian genetic backgrounds has not been evaluated. The retrospective design and the absence of prospective longitudinal monitoring also limit its readiness for clinical implementation. For early-onset GC, Guo and colleagues identified a three-lncRNA signature that yielded an AUC of 0.924 in the training set and 0.911 in validation (Guo X. et al., 2025). It should be noted that early-onset GC is relatively rare, and the sample size of this study was small, which may limit the statistical power and generalizability of the three-marker signature.

Exosomal circRNAs are under active investigation. hsa_circ_0015286 is elevated in GC and declines after surgery (Zheng et al., 2022). Hsa_circ_0065149 offers greater sensitivity and specificity than CEA or CA19-9 (Shao et al., 2020). Plasma exosomal circMAN1A2 yielded an AUC of 0.685 alone and 0.798 when combined with CEA (Shen et al., 2025). The modest incremental benefit over CEA alone (AUC 0.685–0.798) and the lack of comparison with other established biomarkers suggest that this circRNA may be more suitable as an adjunct rather than a standalone diagnostic tool. In gastric signet ring cell carcinoma, plasma exosomal circUBR5 was elevated and distinguished this subtype from conventional adenocarcinoma with an AUC of 0.726 (Jiang et al., 2026).

RsRNAs and tsRNAs have recently entered the exosomal biomarker field. Yang and colleagues profiled the plasma exosomal rs/tsRNA landscape in GC and validated a three-marker panel that distinguished GC from healthy controls with an AUC of 0.91 (sensitivity 80.4%, specificity 87.4%) (Yang et al., 2025). The biological functions of most rs/tsRNAs remain poorly understood, and their diagnostic value reported in this single study has not been independently validated by other groups, highlighting the need for multi-center replication. Detailed information on the above biomarkers is summarized in Table 2.

**TABLE 2:** Exosomes as diagnostic biomarkers for GC:miRNA, lncRNAand circRNA.

Categories	Specific biomarkers	Sample type	Detection techniques	Clinical significance	Reported performance	Ref.
miRNA	miR-502-5p	Serum	qRT-PCR	Inhibit the progression of GC	-	Zhou and Li (2024)
miRNA	Neutrophil-derived exosomal miRNAs	Serum	qRT-PCR	GC diagnostic markers	-	Yu et al. (2024)
miRNA	miR-1246	Plasma	qRT-PCR	Biomarkers and therapeutic targets of GC	AUC 0.65–0.85 across cohorts	Lu et al. (2024)
miRNA	miRNA-124-3p	Gastric fibroblasts	qRT-PCR	Promote the proliferation and migration of gingival fibroblasts	-	Li et al. (2024b)
miRNA	let-7g-3p, miR-10395-3p	Peritoneal lavage fluid	qRT-PCR	Biomarkers for predicting peritoneal metastasis and the efficacy of systemic chemotherapy	-	Luo et al. (2023)
miRNA	Destinex(5 cf-miRNA +5 exo-miRNA:miR-21-3p, miR-21-5p, miR-215-5p, miR-27a-3p, miR-95-3p)	Serum	Small RNA sequencing + qRT-PCR + XGBoost	Early Detection of GC	-	Sui et al. (2025)
lncRNA	lncH19	Serum	qRT-PCR	GC TNM staging	-	Xiao et al. (2024)
lncRNA	HOTAIR	Serum	qRT-PCR	Prediction of GC metastasis	-	Chen et al. (2023a)
lncRNA	LncGC1	-	qRT-PCR	Early biomarkers of neoadjuvant chemotherapy efficacy	-	Guo et al. (2023)
lncRNA	lncAKR1C2	GC Cell	qRT-PCR	Promote GC lymph node metastasis	-	Zhu et al. (2023)
lncRNA	lncRNA-GC1	Plasma	qRT-PCR	Predict and monitor the immunotherapeutic outcomes of patients with GC	Outperforms conventional markers	Wei et al. (2024)
lncRNA	lnc00852	GC Cell	qRT-PCR	Lead to cisplatin resistance	-	Cao et al. (2023)
circRNA	hsa_circ_0015286	Tissue/Plasma /Cancer Cell	qRT-PCR	Promote progression of GC	-	Yoon et al. (2024)
circRNA	has_circ_000200	Serum	qRT-PCR	Assessment of GC progression	-	Huang et al. (2023)
circRNA	CDR1as	Plasma	qRT-PCR	GC diagnosis and prognosis prediction	-	Li et al. (2023c)
circRNA	Circ50547	Plasma	qRT-PCR	GC diagnosis and prognosis prediction	-	Zang et al. (2024)
circRNA	circ_0079439	Plasma	qRT-PCR	A potential biomarker for early and late GC diagnosis	-	Li et al. (2023d)
circRNA	circHIPK3	Serum	qRT-PCR	Promote cisplatin resistance	-	Shang et al. (2023)
circRNA	circMAN1A2	Plasma	qRT-PCR	Promote cancer progression and suppresses T-cell antitumour immunity	AUC 0.685 (alone), 0.798 (+CEA)	Shen et al. (2025)
circRNA	circPTBP3	Plasma	qRT-PCR	Predict the prognosis of gastric cancer	-	Dong et al. (2025b)
rsRNA/tsRNA	S2, S7, S10	Plasma	PANDORA-seq/qRT-PCR	Biomarkers in GC	AUC 0.91, sensitivity 80.4%, specificity 87.4%; early-stage: sensitivity 82.0%, specificity 81.4%	Yang et al. (2025)

### 4.2 Exosomal proteins

Exosomal PD-L1 stands out among protein biomarkers. Elevated circulating exosomal PD-L1 is associated with shorter overall survival. Compared to soluble PD-L1, the exosomal form is more stable, and co-expressed MHC-I molecules on the vesicle surface amplify its immunosuppressive potency (Li H. et al., 2024; Yong et al., 2025).

Additional proteins have shown diagnostic utility. Exosomal MT1-MMP mRNA is upregulated in GC and correlates with metastasis and TNM stage. It yields an AUC of 0.788 alone and 0.821 when combined with CEA (Dong et al., 2019). However, this study included only 70 GC patients, and the combination with CEA provided only a modest improvement, raising questions about its added clinical utility beyond existing serum markers. A five-protein panel consisting of GSN, HP, ORM1, APOA1, and TTR achieved an AUC of 0.80 in a validation set and improved the detection of peritoneal metastasis (Gu et al., 2022). The validation set size was not clearly specified, and the study did not report whether this protein panel could detect peritoneal metastasis earlier than current imaging modalities such as CT or PET-CT, which remains the critical clinical need. A 10-protein signature linked to peritoneal spread has also been described, although its diagnostic performance metrics and validation status were not fully reported in the initial study (Chen et al., 2023b). Detailed information on the above protein markers is summarized in Table 3.

**TABLE 3:** Exosomes as diagnostic biomarkers for GC:protein.

Specific protein biomarkers	Sample type	Detection techniques	Clinical significance	Reported performance	Ref.
THBS2	BMSC-EVs	-	Promote GC development	-	Qi et al. (2023)
HSP60	-	Proteomics	Inhibit apoptosis of infected cells	-	Li et al. (2023e)
ILK1, CD14	Plasma	Mass spectrum	Prediction of GC organ-specific metastasis	-	Zhou et al. (2023)
DACT3-AS1	-	Ultracentrifugation	Diagnostic and therapeutic marker for regulating malignant transformation and oxaliplatin resistance	-	Qu et al. (2023)
DUOXA2, ITGA7, LIMS1, MSRB3, PLCB1,RAB6B, SEMA3C, SMTN, TADA1, TBC1D14	Serum	Centrifugation	Peritoneal metastasis diagnostic markers	-	Chen et al. (2023b)
HER2	Serum	-	Diagnostic and therapeutic biomarker for evaluating HER2 status and predicting trastuzumab efficacy in patients	-	Li et al. (2023b)
PD-L1	Serum	Ultracentrifugation	Prognosis and diagnostic biomarker in GC patients	Shorter OS (prognostic)	Li et al. (2024a)
GSN, HP, ORM1, APOA1, TTR	Serum	Mass spectrum	Biomarkers for GC	AUC 0.80 (validation)	Gu et al. (2022)
PD-L1	Serum	qRT-PCR	Reflect the immunosuppressive status in advanced GC patients	-	Shin et al. (2023)
PKM2	Plasma	qRT-PCR	Biomarker predicting poor prognosis in gastric cancer patients	-	Yuan et al. (2025)
Menin	Plasma	Western blot/Mass spectrum	Promote GC development	-	Wang et al. (2025c)
NOS3	GC Cell	qRT-PCR	Promote liver metastasis	-	Hu et al. (2024)

A closer look at the current biomarker studies reveals shared weaknesses. Most are retrospective, single-center efforts with cohort sizes below 100. Independent external validation remains rare. Reported AUCs for the same molecule can span a wide range. For example, exosomal miR-1246 yields AUCs from 0.65 to 0.85 across different cohorts and detection methods. Pre-analytical variables, including blood collection protocols, storage duration, and the exosome isolation technique, are not standardized and directly affect reproducibility. Multicenter prospective trials with harmonized protocols are needed before any of these candidates can be considered for clinical use. Furthermore, the majority of these biomarker studies have been conducted in surgical or oncology center populations, where the prevalence of GC is artificially elevated. Their performance in primary care or community-based screening settings, where the true clinical need lies, remains virtually unexplored. The cost-effectiveness and patient acceptance of multi-analyte exosomal panels also require formal health economic evaluation before they can be adopted into routine practice.

### 4.3 Technological advances and clinical challenges in exosome diagnosis

Aptamer-functionalized microfluidic chips can now capture exosomes with high sensitivity and capture rates exceeding 90% of target vesicles, bypassing many limitations of traditional ultracentrifugation. Yu and colleagues developed an integrated chip for analyzing neutrophil-derived exosomes that enables simultaneous detection of surface proteins and internal miRNAs using 10 μL of serum within 4 hours. In a cohort of 71 healthy controls, 50 patients with benign gastric disease, and 67 GC patients, the neutrophil-derived exosome marker panel achieved an AUC of 0.891 for distinguishing GC from healthy controls (Yu et al., 2025). Despite the analytical sensitivity achieved by these microfluidic platforms, most have been tested on small, single-center cohorts and require validation in large, multicenter settings. The transition from proof-of-concept device to commercially available, regulatory-approved diagnostic kit remains a substantial hurdle.

For biomarker screening, machine learning algorithms have significantly improved diagnostic accuracy. Sui and colleagues employed the XGBoost algorithm to establish the Destinex panel and confirmed its stability in an independent validation cohort (Sui et al., 2025). In EV-DNA analysis, the TEMPT technique developed by Lin et al. enables high-quality methylation profiling from approximately 1.5 mL of plasma (Lin et al., 2025).

DNA nanotechnology-based platforms have provided a new paradigm for ultrasensitive exosomal nucleic acid detection. Guo et al. developed BEISA, a dual-modal *in situ* analyzer for exosomal circRNA that integrates fluorescence and electrochemical signal output (Guo Y. et al., 2025). In an analysis of 75 clinical specimens, a model integrating four circRNAs distinguished GC from healthy controls with AUCs ranging from 0.925 to 1.00 and differentiated early-stage from advanced GC with an accuracy of 85.0%. He et al. devised a SERS sensor based on CRISPR/Cas13a-triggered DNA walker amplification for measuring GC-associated exosomal miR-106a, which discriminated 20 GC patients from 10 healthy controls with an AUC of 0.985 (He X. et al., 2025).

In exosomal protein analysis, Zhang et al. reported a SERS detection platform using multivalent aptamer-tetrahedral DNA conjugates coupled with catalytic hairpin assembly, achieving a detection limit of 2.98 × 10^3^ particles/mL and a reported accuracy of 100% in distinguishing GC patients from healthy individuals (Zhang J. et al., 2025). The same group further refined the strategy by adopting branched hybridization chain reaction, yielding the triApt-bHCR-SERS platform with a detection limit of 0.39 particles/μL and an AUC of 0.987 in a preliminary clinical evaluation (Liu X. et al., 2025). The performance characteristics of these detection platforms are summarized in Table 4.

**TABLE 4:** Exosome detection technology platform.

Platform	Detection target	Detection limit	Time	AUC	Sample size	Ref.
Integrated microfluidic chip (CD66b^+^ NEV)	Neutrophil-derived exosome miRNAs and surface proteins	90% capture efficiency at 0.5 μL/min	∼4 h	0.891 (GC vs. healthy)	71 healthy, 50 benign, 67 GC	Yu et al. (2025)
Destinex (XGBoost)	5 cell-free +5 exosomal miRNAs	—	—	0.948 (validation)	809 specimens	Sui et al. (2025)
TEMPT (whole-genome methylation)	EV-DNA methylation	Sub-nanogram input	—	0.80 (validation)	58 GC and gastric polyps	Lin et al. (2025)
BEISA (DNA framework-guided HCR)	Exosomal circRNA (4-circRNA panel)	8.70 aM	—	0.925–1.00 (GC vs. healthy); 85.0% accuracy (early vs. advanced)	75 clinical specimens	Guo et al. (2025b)
CRISPR/Cas13a SERS sensor	Exosomal miR-106a	53.16 aM	80 min	0.985	20 GC, 10 healthy	He et al. (2025b)
Aptamer-tetrahedral DNA SERS	Exosomal MUC1/CD63	2.98 × 10^3^ particles/mL	—	100% accuracy (GC vs. healthy)	—	Zhang et al. (2025a)
triApt-bHCR-SERS	Exosomal MUC1/CD63	0.39 particles/μL	—	0.987	—	Liu et al. (2025b)

### 4.4 Methodological heterogeneity and standardization

A critical barrier to the clinical translation of exosome-based biomarkers is the lack of methodological consensus across studies. Exosome isolation remains the largest source of variability. Ultracentrifugation, the most widely used method, yields high purity but co-pellets non-exosomal particles and suffers from poor reproducibility across laboratories. Precipitation-based commercial kits recover higher yields but at the cost of purity, while size-exclusion chromatography offers a balance between purity and vesicle integrity but is less amenable to high-throughput processing. Microfluidic and affinity-based capture methods have shown improved selectivity, yet their reliance on specific surface markers introduces capture bias toward particular exosome subpopulations. These differences in isolation technique directly affect the molecular composition of the resulting exosome preparation and contribute to the wide range of reported AUCs observed for individual biomarkers.

Characterization methods are similarly non-uniform. Nanoparticle tracking analysis and tunable resistive pulse sensing measure particle concentration and size distribution but cannot distinguish exosomes from non-exosomal particles of similar dimensions. Flow cytometry, particularly with high-sensitivity instruments, enables single-vesicle phenotyping but is limited by antibody availability and instrument standardization. The absence of a consensus reference material for exosome calibration compounds these difficulties. Finally, reporting practices vary widely. Minimal information for studies of extracellular vesicles guidelines, published in 2018 and updated in 2024, provide a framework for transparent reporting of isolation, characterization, and functional experiments (Théry et al., 2018; Welsh et al., 2024). However, adherence remains inconsistent. Broader adoption of these standards, combined with inter-laboratory ring trials and the development of certified reference materials, will be essential for transforming exosome-based diagnostics from exploratory findings into clinically validated tools.

Beyond standardization, the specificity of candidate biomarkers still requires confirmation in larger, multicenter prospective cohorts. Recent investigations, including the Destinex study and a four-lncRNA signature, provide exemplary paradigms, underscoring that panels of multiple analytes, rather than single molecular entities, represent a more viable path forward for liquid biopsy. Looking ahead, the establishment of international consensus guidelines, the development of automated microfluidic chip-based detection platforms, and the integration of machine learning algorithms will be pivotal in propelling exosome diagnostics from the bench to the bedside.

Ultimately, the adoption of exosomal biomarkers in clinical practice will depend not only on technological innovation but also on the establishment of a regulatory framework that defines acceptable performance thresholds, quality control standards, and clinical utility criteria for these novel analytes. The process is still in its infancy.

## 5 Therapeutic potential

In the field of therapy, exosomes demonstrate dual translational value: they can serve as both therapeutic targets and drug delivery vehicles.

### 5.1 Therapeutic targets

#### 5.1.1 Targeted exosomes generation and release

Suppressing the biogenesis and release of exosomes constitutes an effective strategy for abrogating their functions at the source. The broad-spectrum exosome inhibitor GW4869 has demonstrated therapeutic potential across diverse tumor models by modulating the immunosuppressive tumor microenvironment. A more specific approach involves targeting key enzymes essential for exosome biogenesis, such as RAB27A or nSMase2. SiRNA targeting these molecules reduce immunosuppressive cargo in ascitic fluid by 40%–60%, thereby helping to alleviate T-cell anergy (Zhang et al., 2026). In the context of the hypoxic microenvironment characteristic of GC, proton pump inhibitors have been found to restrain malignant progression by inhibiting exosome release and regulating the HIF-1α-FOXO1 axis (Guan et al., 2017). It is important to note that broad-spectrum inhibitors such as GW4869 also affect other sphingomyelinase-dependent processes beyond exosome biogenesis, raising concerns about off-target effects. Moreover, most of these inhibition strategies have been validated only *in vitro* or in subcutaneous xenograft models. Their efficacy and safety in orthotopic GC models, which better mimic the human disease, have not been systematically examined.

### 5.1.2 Targeting exosomal cargo

PD-L1 stands among the most intensely investigated therapeutic targets at present. Multiple interventional strategies have been validated for their capacity to modulate either the abundance or the functionality of exosomal PD-L1. Knocking out LSD1 reduces exosomal PD-L1 levels and restores T-cell function (Shen D.-D. et al., 2022). The small molecule EP16 curbs exosomal PD-L1 secretion, thereby boosting T-cell activation and working in concert with anti-PD-1 therapy (Sun et al., 2024). Melatonin alters PD-L1 levels in macrophages by modulating exosomal miRNA cargo (Wang K. et al., 2023). Additionally, an extract of the traditional Chinese formula Jianpi Yangzheng Fang blunts the exosomal PD-L1-driven expansion of myeloid-derived suppressor cells (MDSCs) (Chen et al., 2023c). Although these interventions demonstrate mechanistic plausibility, their therapeutic efficacy has been evaluated primarily in immunodeficient or single-cell-line models. The impact of these strategies on the broader immune ecosystem, including potential disruption of normal immune homeostasis, has not been thoroughly assessed. Furthermore, the active components in herbal formulations such as Jianpi Yangzheng Fang are complex and not fully characterized, complicating mechanistic interpretation and quality control for translational development.

**FIGURE 7:**
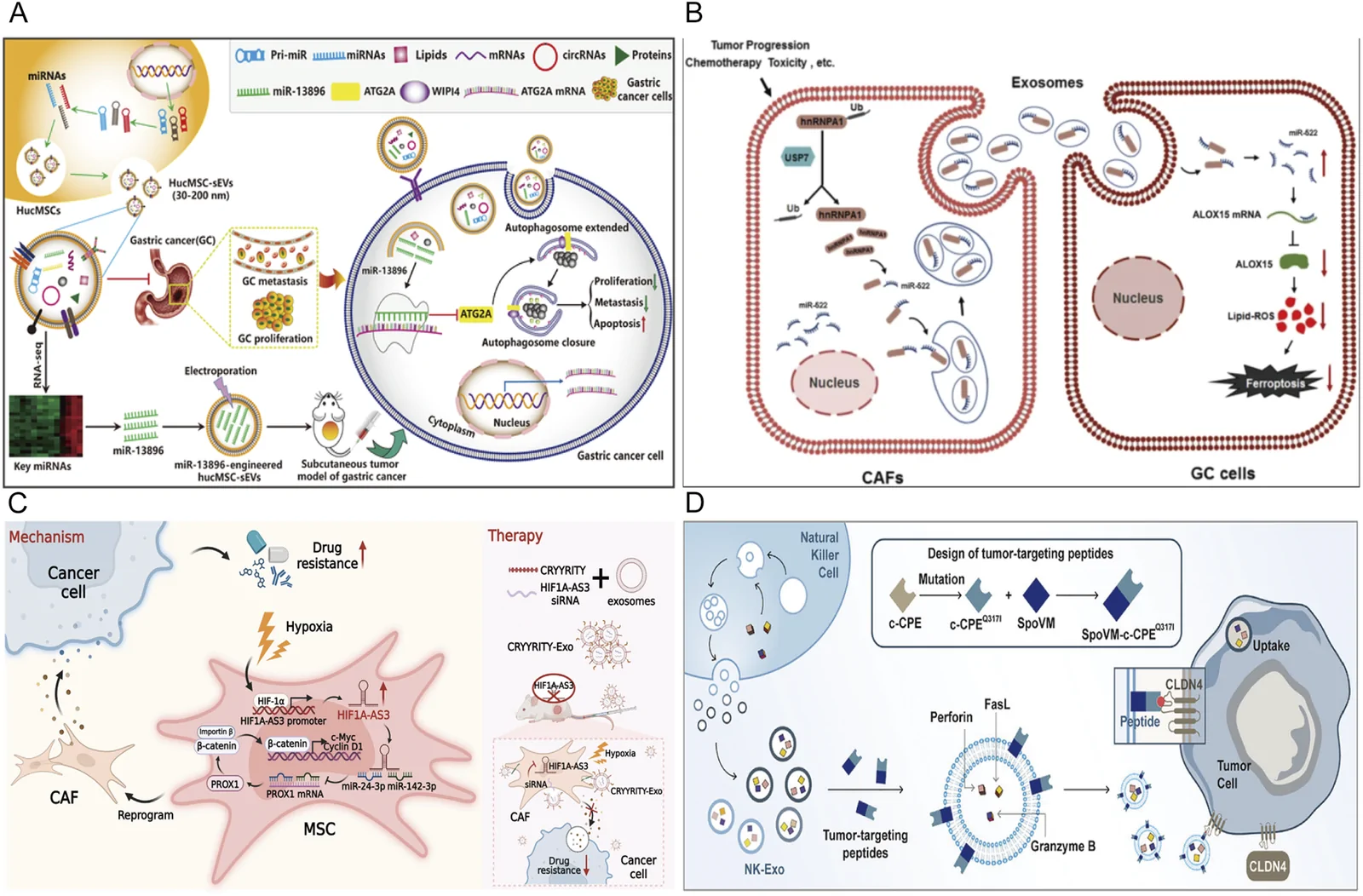
Therapeutic potential of natural and engineered exosomes in GC. **(A)** HucMSC-sEVs deliver miR-13896 to GC cells, where it blocks ATG2A-mediated autophagy and inhibits tumor progression (Wu et al., 2024). From Wu, P. et al., Biomater. Res. 28, 0119 (2024). DOI: 10.34133/bmr.0119. Reproduced under the terms of the CC BY 4.0 license. **(B)** CAF-derived miR-522 blocks ferroptosis and drives acquired chemoresistance in GC (Zhang H. et al., 2020). From Zhang, H. et al., Mol. Cancer 19, 43 (2020). DOI: 10.1186/s12943-020-01168-8. Reproduced under the terms of the CC BY 4.0 license. **(C)** Proposed model illustrating how HIF1A-AS3 reprograms MSCs and contributes to chemoresistance in GC (Xu et al., 2025). From Xu, J. et al., Drug Resist. Updat. 82, 101275 (2025). DOI: 10.1016/j.drup.2025.101275. Reprinted with permission from Elsevier. **(D)** Design of NK-sEV-SpoVM-c-CPEQ317I (NESC) and its tumor-targeted delivery in GC (Dong A. et al., 2025). From Dong, A. et al., J. Extracell. Vesicles 14, e70200 (2025). DOI: 10.1002/jev2.70200. Reproduced under the terms of the CC BY 4.0 license.

Non-coding RNAs likewise represent critical cargoes amenable to targeted intervention. Silencing or inhibiting the expression of miR-10b-5p attenuates GC cell proliferation (Yan et al., 2021). The administration of exosome-mediated miR-374a-5p inhibitors reduces resistance to oxaliplatin (Ji et al., 2019). Furthermore, targeting miR-522 within exosomes secreted by CAFs promotes ferroptosis and enhances chemosensitivity (Zhang H. et al., 2020) (Figure 7B).

### 5.2 Engineered exosomes as therapeutic agents

As drug delivery vehicles, exosomes possess distinctive advantages conferred by their natural origin, including low immunogenicity, minimal toxicity, surface enrichment of biomolecules that serve as recognition signals for precise targeting, and a lipid bilayer architecture that safeguards encapsulated cargo from degradation (Pan et al., 2025).

#### 5.2.1 Therapeutic potential of natural exosomes

Small EVs (sEVs) from umbilical cord mesenchymal stem cells carry anti-cancer miRNAs naturally. One of them, miR-13896, inhibits GC cell growth, triggers apoptosis, and blocks metastasis by disrupting ATG2A-driven autophagy (Wu et al., 2024) (Figure 7A). While these findings are encouraging, the study relied on subcutaneous xenograft models, which do not recapitulate the immunosuppressive gastric microenvironment. The natural heterogeneity of MSC-derived exosome populations also poses challenges for batch-to-batch consistency in large-scale production.

Exosomes isolated from malignant ascites and then heat-treated can accelerate dendritic cell maturation and provoke tumor-specific cytotoxic T cell responses, making them a potential cell-free vaccine (Zhong et al., 2011). This approach has only been tested in preclinical mouse models, and the feasibility of generating sufficient quantities of heat-treated exosomes from patient ascites for clinical application, as well as the potential immunogenicity of repeated dosing, has yet to be addressed.

Bone marrow-derived MSC exosomes (hBM-MSC-exos) have context-dependent effects. Under normal conditions, they promote EMT and enhance GC cell proliferation, migration and invasion. However, when hBM-MSCs are primed with oxaliplatin, the secreted exosomes gain an opposite function. Oxaliplatin upregulates miR-424-3p in these exosomes, and the resulting Oxa-MSC-exos suppress EMT, reduce clonogenicity, migration and invasion, and slow tumor growth in xenograft mice. The mechanism involves exosomal miR-424-3p directly targeting and downregulating the pro-tumorigenic factor RHOXF2. Thus, chemotherapy-primed BM-MSC exosomes can act as natural anti-cancer agents, even though their naive counterparts are tumor-promoting (Shen et al., 2024). The finding that chemotherapy-primed MSCs switch from pro-tumor to anti-tumor is intriguing, but whether this phenomenon is specific to oxaliplatin or generalizable to other chemotherapeutic agents remains unknown. Moreover, the fraction of MSCs that successfully incorporate miR-424-3p and the long-term stability of these engineered vesicles in circulation have not been determined.

Immune cell-derived exosomes also play complex roles. M2-polarized tumor-associated macrophages (TAMs) release exosomes enriched in miR-194. These exosomes are taken up by GC cells, where miR-194 directly targets PTEN, activates PI3K/AKT signaling, and upregulates Bcl-2. Consequently, they reduce cisplatin-induced apoptosis and confer chemoresistance in both murine and human GC cells. *In vivo*, intratumoral injection of miR-194 promotes tumor growth and weakens cisplatin efficacy (Zhou et al., 2025). Hence, TAM exosomes can act as mediators of drug resistance, representing a therapeutic target rather than a direct treatment.

Natural exosomes can also be pharmacologically targeted. Zhang et al. showed that the herbal formula Jianpi Yangzheng decoction (JPYZ) suppresses gastric cancer invasion and migration by modulating TAM-derived exosomes. JPYZ reduces the level of miR-513b-5p in these exosomes. When taken up by GC cells, exosomal miR-513b-5p binds to the 3′-UTR of PTEN, downregulates it, and activates AKT/mTOR signaling to promote invasion and migration. By lowering exosomal miR-513b-5p, JPYZ restores PTEN expression, inhibits AKT/mTOR signaling, reverses EMT, and suppresses tumor growth in xenograft models. This work highlights that natural exosomes from immune cells can be modulated by herbal medicines to achieve anti-cancer effects, pointing to a new strategy of targeting pathogenic exosomal miRNAs (Zhang R. et al., 2024). The multi-component nature of JPYZ makes it difficult to attribute the observed effects solely to miR-513b-5p modulation, and the pharmacokinetics and bioavailability of its active ingredients in humans are not well established.

#### 5.2.2 Engineered exosomes as drug delivery agents

Targeted delivery of nucleic acid therapeutics constitutes one of the foremost applications of engineered exosomes. Exosome-mediated delivery of siRNA against hepatocyte growth factor suppresses GC cell proliferation (Zhang H. et al., 2018). Delivery of c-Met siRNA reverses cisplatin resistance (Zhang Q. et al., 2020), and administration of anti-miR-214 attenuates platinum-based chemoresistance (Zheng et al., 2025; Wang X. et al., 2018). These siRNA-loaded exosome studies were predominantly conducted in subcutaneous xenograft models and used electroporation for loading, a method known to cause RNA aggregation and reduced bioactivity. The translation of these approaches to patients will require development of more efficient and scalable loading techniques, along with rigorous pharmacokinetic and toxicological assessment in large animal models. Exosomal transfer of miR-29b inhibits peritoneal metastasis (Kimura et al., 2023). For chemotherapeutic drug delivery, DE532-engineered exosomes loaded with 17-DMAG achieve precise targeting of GC cells while mitigating adverse effects (Park et al., 2024). In HER2-positive GC, tumor-derived exosomes capture and shuttle trastuzumab-emtansine to target tissues (Barok et al., 2018). The targeting efficiency and therapeutic window of these drug-loaded exosomes have not been benchmarked against clinically approved nanoparticle formulations such as liposomes or antibody-drug conjugates. Furthermore, large-scale GMP-grade production remains a significant bottleneck.

Several engineered platforms have been developed to target the tumor microenvironment. Xu et al. constructed integrin α5 peptide-modified exosomes for delivery of HIF1A-AS3 siRNA to tumor-resident MSCs and CAFs. When combined with oxaliplatin, this system suppressed tumor growth and induced apoptosis (Xu et al., 2025) (Figure 7C). While this combination showed synergistic effects *in vitro* and *in vivo*, the study did not evaluate potential off-target accumulation in normal organs, and the long-term toxicity of repeated siRNA-exosome administration was not assessed. The therapeutic benefit over oxaliplatin alone in orthotopic models, which more closely mimic the clinical scenario, was modest. Dong et al. devised NESC, an NK cell-derived exosome platform conjugated with a CLDN4-targeting peptide. NESC elevated intracellular ROS levels and potentiated radiotherapy-induced DNA double-strand breaks. In patient-derived organoids and orthotopic xenograft models, NESC combined with radiotherapy markedly suppressed tumor growth and prolonged survival (Dong A. et al., 2025) (Figure 7D). The patient-derived organoid and orthotopic models used in this study represent a significant advance over conventional xenografts. Nevertheless, the safety profile of NESC, particularly its potential to activate NK cells in off-target tissues and induce cytokine release syndrome, requires careful evaluation before clinical translation. The manufacturing complexity of conjugating targeting peptides to NK-derived exosomes also poses scalability challenges.

Tang et al. engineered hybrid vesicles, termed Neu/MSC-sEVs, by fusing hucMSC-sEVs with neutrophil membranes. These vesicles retain the antitumor effector protein Pentraxin 3 from hucMSC-sEVs and acquire chemokine receptors and CD47 from the neutrophil membrane, achieving enhanced tumor cell uptake, prolonged circulatory half-life, and preferential enrichment within neoplastic tissue. In tumor-bearing mice, Neu/MSC-sEVs showed markedly smaller tumors than native hucMSC-sEVs (Tang Y. et al., 2025). The key features of these engineered exosome platforms are summarized in Table 5. Although the hybrid membrane engineering strategy enhances circulation time and tumor uptake, the immunogenicity of the inserted neutrophil membrane proteins in repeated dosing regimens has not been tested. The long-term fate and degradation profile of these hybrid vesicles *in vivo* remain largely unknown.

**TABLE 5:** Engineered exosome delivery system.

Engineering strategy	Targeting modification	Cargo	Model	Key finding	Ref
Unmodified exosomes	None	HGF siRNA	*In vitro*	Suppressed GC cell proliferation	Zhang et al. (2018a)
Unmodified exosomes	None	c-Met siRNA	*In vitro*	Reversed cisplatin resistance	Zhang et al. (2020b)
Unmodified exosomes	None	Anti-miR-214	*In vitro*/*in vivo*	Attenuated platinum-based chemoresistance	Zheng et al. (2025), Wang et al. (2018c)
Unmodified exosomes	None	miR-29b	*In vivo*	Inhibited peritoneal metastasis	Kimura et al. (2023)
DE532-engineered exosomes	DE532 peptide	17-DMAG	*In vitro*	Precise GC cell targeting, reduced adverse effects	Park et al. (2024)
Tumor-derived exosomes	HER2 (intrinsic)	Trastuzumab-emtansine	*In vitro*/*in vivo*	Targeted drug delivery to HER2-positive GC	Barok et al. (2018)
Integrin α5 peptide-modified exosomes	Integrin α5 peptide	HIF1A-AS3 siRNA	*In vitro*/*in vivo* (xenograft)	Suppressed tumor growth when combined with oxaliplatin	Xu et al. (2025)
NESC (NK cell-derived exosomes with CLDN4 peptide)	CLDN4-targeting peptide (c-CPEQ317I), SpoVM	None (ROS elevation)	Patient-derived organoids, PDOX	Potentiated radiotherapy, prolonged survival	Dong et al. (2025a)
Neu/MSC-sEVs (MSC exosome-neutrophil membrane hybrid)	Chemokine receptors, CD47 (from neutrophil membrane)	Pentraxin 3	*In vivo* (xenograft)	Enhanced tumor uptake, prolonged circulation, reduced tumor burden	Tang et al. (2025b)

Despite these advances, exosome-based therapies face a set of practical barriers that limit their clinical translation. Scaling production under GMP conditions remains difficult. Ultracentrifugation, the most widely used isolation method, is not designed for large-scale manufacturing. Cargo loading, whether by electroporation, sonication, or co-incubation, yields batch-to-batch variability that complicates quality control. Targeting specificity poses an additional challenge: even when surface ligands improve tumor cell recognition, systemically administered exosomes still accumulate predominantly in the liver and spleen, reducing the effective dose at the tumor site. Biodistribution and long-term fate have not been systematically tracked in orthotopic GC models. Safety concerns include the potential transfer of oncogenic molecules from producer cells and the risk that repeated dosing of engineered vesicles, especially those carrying foreign targeting peptides, may provoke neutralizing antibodies and compromise therapeutic efficacy. Furthermore, most therapeutic studies have been conducted in subcutaneous xenograft models; validation in orthotopic or spontaneous tumor models, which better recapitulate the human GC microenvironment, is scarce. Moreover, strategies aimed at inhibiting exosome biogenesis or targeting exosomal cargo face an additional layer of complexity: the molecular machinery that produces pathogenic exosomes is often essential for normal cellular physiology, and the active components of herbal formulations remain poorly defined, complicating both mechanistic interpretation and quality control. The regulatory framework for exosome-based products also remains undefined, as these vesicles do not fit neatly into existing categories for biologics or cell-based therapies. Until these manufacturing, targeting, safety, and clinical validation hurdles are addressed in parallel, the gap between preclinical promise and clinical application will persist. Beyond these technical and biological hurdles, the economic viability of exosome-based therapeutics remains an underappreciated yet critical barrier. The production cost per dose for GMP-grade engineered exosomes is currently estimated to be substantially higher than that of monoclonal antibodies or conventional nanoparticles, and the reimbursement landscape for such novel biologics is entirely uncharted. The lack of established potency assays and release criteria further complicates regulatory approval. Finally, the clinical utility of exosome-based therapies must be demonstrated not only in molecularly selected patient subsets but also in head-to-head comparisons with existing standard-of-care regimens. Without such comparative evidence, it will be difficult to justify the added complexity and cost of exosome-based approaches in routine oncology practice.

## 6 Discussion

**FIGURE 8:**
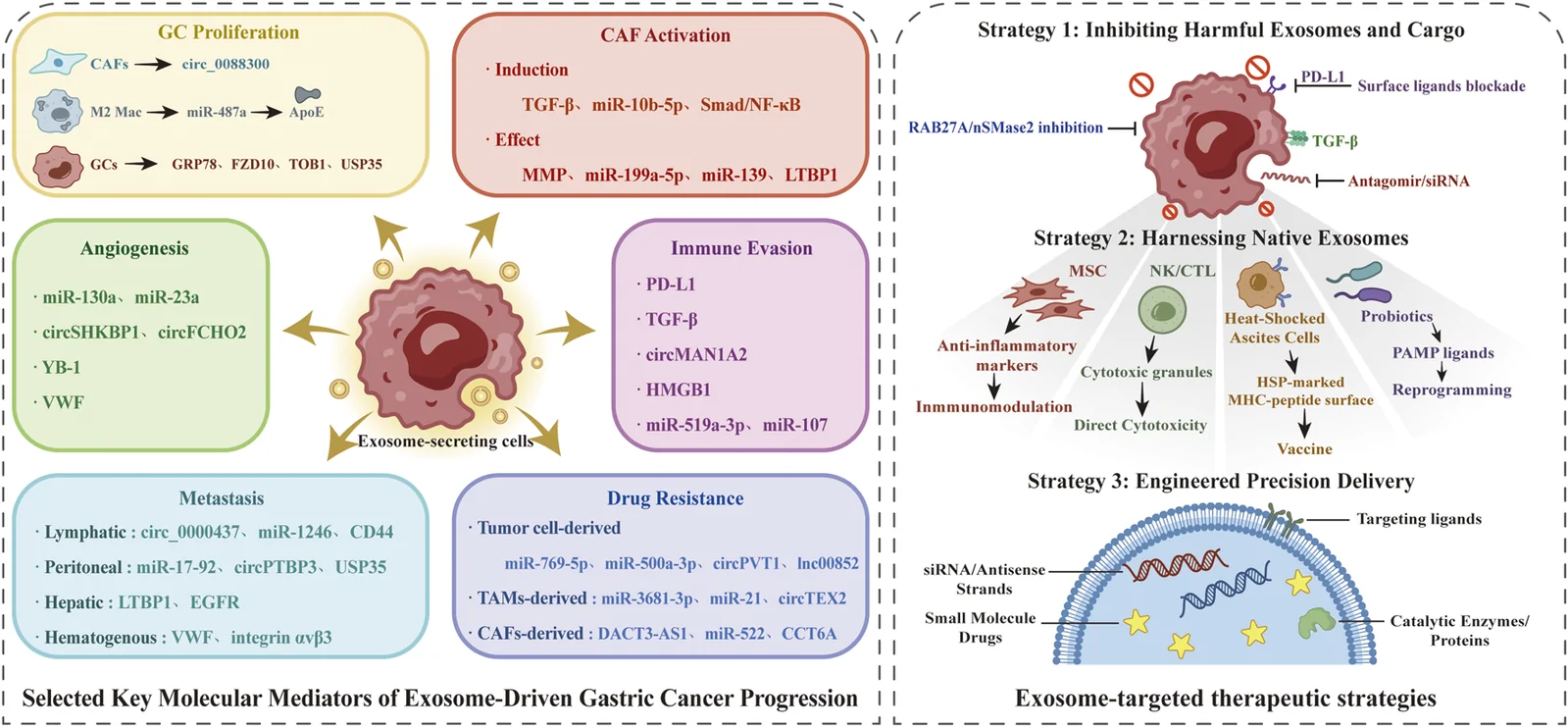
Key exosomal mediators in gastric cancer progression and hierarchical therapeutic strategies.

This review has examined the roles of exosomes across six dimensions of GC progression, from *H. pylori* driven inflammation to pre-metastatic niche conditioning and therapeutic resistance. Several themes emerge from this review (Figure 8).

First, a consistent observation across proliferation, immune evasion, and metastasis is the convergence of diverse exosomal cargoes onto a limited set of core signaling pathways, including PI3K/AKT, MAPK/ERK, TGFβ/Smad, and NFκB. This redundancy has two implications. On one hand, it explains why targeting a single exosomal molecule in preclinical models often yields modest effects and suggests that interfering with exosome biogenesis or common downstream nodes may be more therapeutically rational than pursuing individual cargoes. On the other hand, it raises a caution: many reported molecules may represent generic stress responsive transcripts that are packaged into exosomes and amplified under *in vitro* conditions rather than disease specific drivers. Distinguishing causative drivers from passenger cargoes requires systematic loss of function screens coupled with *in vivo* rescue experiments, an approach that remains uncommon in current literature.

Second, the diagnostic performance of exosomal non-coding RNAs and proteins in retrospective case-control studies appears promising, with some AUC values exceeding 0.95. However, the gap between discovery and clinical deployment remains substantial. Most studies are single-center, retrospective, and underpowered, with cohort sizes below 100. The same miRNA, such as miR-1246, can yield AUCs ranging from 0.65 to 0.85 depending on isolation methods and cohort composition, indicating that pre-analytical standardization constitutes the current rate-limiting step. Moreover, virtually all diagnostic studies have been conducted in surgical or oncology populations with artificially elevated disease prevalence. Their performance in primary care screening settings, where GC prevalence is low, has not been tested. Large prospective multicenter screening trials are required before exosomal biomarkers can be considered for clinical use.

Third, engineered exosomes offer certain advantages over synthetic nanoparticles, including natural tropism and low immunogenicity. However, the preclinical literature is dominated by subcutaneous xenograft models that do not recapitulate the immunosuppressive and desmoplastic GC microenvironment. Orthotopic and patient-derived organoid models remain infrequently used. Manufacturing and regulatory hurdles are also substantial. GMP-grade exosome production is expensive and poorly scalable. Electroporation-based loading can damage RNA, and systemically administered exosomes accumulate predominantly in the liver and spleen, with only a small fraction reaching the tumor. Head-to-head comparisons with clinically approved nanocarriers are lacking, and the projected cost per dose for engineered exosomes exceeds that of monoclonal antibodies. Reimbursement pathways have not been established.

Fourth, several contradictions in the literature merit acknowledgment. miR-21 has been reported to promote chemoresistance in some studies but to exert tumor-suppressive functions in others, likely due to context-dependent target repertoires. MSC-derived exosomes are tumor-promoting under normal conditions but become anti-tumor after chemotherapy priming, a dichotomy that requires mechanistic dissection. The relative contribution of exosomal PD-L1 versus surface PD-L1 on tumor cells to systemic immunosuppression remains unclear. Resolving these contradictions will require standardized reference materials, inter-laboratory ring trials, and single-vesicle resolution profiling.

In conclusion, exosome biology in GC has progressed from a descriptive phase to one with translational potential. Realizing this potential requires several shifts: from cataloguing molecules to prioritizing the most robust and reproducible mechanisms, from retrospective discovery to prospective validation, and from preclinical proof-of-concept to rigorous engineering and economic assessment. These priorities should guide future investigations in the field.

## 7 Conclusion and future perspectives

Exosomes function as central messengers within the GC microenvironment, coordinating tumor proliferation, immune evasion, angiogenesis, stromal remodeling, metastasis, and therapeutic resistance through an interconnected molecular network. From *H. pylori* infection to the conditioning of pre-metastatic niches and the dissemination of drug-resistant clones, exosomes are involved at every stage of disease progression. This review has examined the mechanisms by which exosomal cargoes drive each of these processes, evaluated their potential as liquid biopsy biomarkers, and assessed strategies that either target exosomes or employ them as therapeutic delivery vehicles.

Despite substantial progress, several fundamental barriers impede the clinical translation of exosome-based applications in GC. Exosomes exhibit remarkable molecular heterogeneity, even when derived from a single cell type, making it difficult to distinguish functionally causative cargoes from passive passengers. The lack of standardized protocols for exosome isolation, quantification, and cargo analysis across laboratories remains the single greatest obstacle to cross-study reproducibility.Ultracentrifugation, precipitation, and size-exclusion chromatography each yield preparations with distinct purity and composition profiles, directly contributing to the wide range of reported AUCs for individual biomarkers. The overwhelming majority of biomarker studies are single-center, retrospective analyses with cohort sizes below 100. Performance in real-world screening populations—where GC prevalence is low-has not been tested.

Addressing these bottlenecks should take precedence over more distant translational aspirations. We propose the following priorities for the next phase of exosome research in GC. Integrating exosome-based liquid biopsy with spatial transcriptomics may enable simultaneous profiling of tumor tissue and systemic exosomal cargo, providing a more complete picture of disease status. Similarly, the safe development of engineered exosome therapeutics requires systematic evaluation of biodistribution, immunogenicity, and long-term fate in large animal models before clinical testing can be responsibly initiated. These efforts will be complemented by advances in single-cell sequencing and spatial transcriptomics, which together will help construct a dynamic atlas of exosome-mediated intercellular communication.

Critically, the field must also address its methodological foundations. We advocate for the adoption of a tiered evidence reporting system in future exosome research, in which findings validated across multiple independent cohorts or interventional models are clearly distinguished from single-study observations. This should be accompanied by open-access sharing of standardized isolation protocols and raw data, enabling robust cross-study comparisons. Through such disciplined practices, the exosome field in GC can achieve the level of clinical credibility that has propelled cfDNA-based liquid biopsy and immune checkpoint inhibitors into routine practice.

In the longer term, once these foundational barriers in standardization, validation, and manufacturing are addressed, more ambitious applications may become feasible. Engineering exosomes that sense microenvironmental cues and release therapeutic payloads on demand, or leveraging patient-derived exosomes to inform both molecular subtyping and the design of tailored exosome-based treatments, represent exciting but distant goals. Continued progress along the directions outlined here holds considerable potential to advance not only the diagnosis and treatment of GC, but also the broader field of exosome biology in other solid malignancies.

## Glossary

2,3-BdpM: 2,3-diphosphoglycerate-dependent phosphoglycerate mutase
AUC: area under the curve
bEVs: bacterial extracellular vesicles
BGN: Biglycan
bHCR: branched hybridization chain reaction
CAFs: Cancer‑associated fibroblasts
CEHs: CAF-educated HSCs
ceRNA: competing endogenous RNA
CTL: cytotoxic T lymphocyte
DCs: Dendritic cells
EBV: Epstein–Barr virus
EMT: epithelial-mesenchymal transition
EOGC: early‑onset GC
EVs: extracellular vesicles
FPR1: formyl peptide receptor 1
GC: gastric cancer
gcEVs: GC cell-derived exosomes
GRP78: Glucose-regulated protein 78
hBM‑MSC‑exos: Bone marrow‑derived MSC exosomes
HSCs: hepatic stellate cells
JPYZ: Jianpi Yangzheng decoction
MDSCs: myeloid‑derived suppressor cells
MMPs: matrix metalloproteinases
MMT: mesothelial-to-mesenchymal transition
MVB: multivesicular body
NK: natural killer
NK‑sEVs: natural killer cell‑derived small extracellular vesicles
OMVs: outer membrane vesicles
PDOX: patient‑derived GC organoids and organoid‑derived xenograft
ROS: reactive oxygen species
rsRNAs: Ribosomal RNA-derived small RNAs
sEVs: Small EVs
TAMs: Tumor‑associated macrophages
TANs: Tumor-associated neutrophils
TLRs: Toll‑like receptors
tsRNAs: transfer RNA-derived small RNAs
VWF: von Willebrand factor
YB‑1: Y‑box binding protein 1
